# Temporal Changes in BEXSERO^®^ Antigen Sequence Type Associated with Genetic Lineages of *Neisseria meningitidis* over a 15-Year Period in Western Australia

**DOI:** 10.1371/journal.pone.0158315

**Published:** 2016-06-29

**Authors:** Shakeel Mowlaboccus, Timothy T. Perkins, Helen Smith, Theo Sloots, Sarah Tozer, Lydia-Jessica Prempeh, Chin Yen Tay, Fanny Peters, David Speers, Anthony D. Keil, Charlene M. Kahler

**Affiliations:** 1 Marshall Centre for Infectious Disease Research and Training, School of Pathology and Laboratory Medicine, University of Western Australia, Perth, Australia; 2 Public Health Microbiology, Forensic and Scientific Services, Health Support Queensland Department of Health, Brisbane, Australia; 3 Sir Albert Sakzewski Virus Research Centre, Queensland Paediatric Infectious Diseases Laboratory, Royal Children’s Hospital, Brisbane, Australia; 4 Department of Microbiology, QEII Medical Centre, PathWest Laboratory Medicine WA, Perth, Australia; 5 Department of Microbiology, Princess Margaret Hospital for Children, PathWest Laboratory Medicine WA, Perth, Australia; 6 Telethon Kids Institute, Perth, WA, Australia; Naval Research Laboratory, UNITED STATES

## Abstract

*Neisseria meningitidis* is the causative agent of invasive meningococcal disease (IMD). The BEXSERO^®^ vaccine which is used to prevent serogroup B disease is composed of four sub-capsular protein antigens supplemented with an outer membrane vesicle. Since the sub-capsular protein antigens are variably expressed and antigenically variable amongst meningococcal isolates, vaccine coverage can be estimated by the meningococcal antigen typing system (MATS) which measures the propensity of the strain to be killed by vaccinated sera. Whole genome sequencing (WGS) which identifies the alleles of the antigens that may be recognised by the antibody response could represent, in future, an alternative estimate of coverage. In this study, WGS of 278 meningococcal isolates responsible for 62% of IMD in Western Australia from 2000–2014 were analysed for association of genetic lineage (sequence type [ST], clonal complex [cc]) with BEXSERO^®^ antigen sequence type (BAST) and MATS to predict the annual vaccine coverage. A hyper-endemic period of IMD between 2000–05 was caused by cc41/44 with the major sequence type of ST-146 which was not predicted by MATS or BAST to be covered by the vaccine. An increase in serogroup diversity was observed between 2010–14 with the emergence of cc11 serogroup W in the adolescent population and cc23 serogroup Y in the elderly. BASTs were statistically associated with clonal complex although individual antigens underwent antigenic drift from the major type. BAST and MATS predicted an annual range of 44–91% vaccine coverage. Periods of low vaccine coverage in years post-2005 were not a result of the resurgence of cc41/44:ST-146 but were characterised by increased diversity of clonal complexes expressing BASTs which were not predicted by MATS to be covered by the vaccine. The driving force behind the diversity of the clonal complex and BAST during these periods of low vaccine coverage is unknown, but could be due to immune selection and inter-strain competition with carriage of non-disease causing meningococci.

## Introduction

*Neisseria meningitidis* (the meningococcus) is the causative agent of invasive meningococcal disease (IMD). IMD is characterised by meningitis and/or fatal septicaemia. The highest incidence of IMD occurs in infants < 12 months of age and in young adults (15–24 years) [1]. Meningococci expressing capsule serogroups A, B, C, W, Y and X are most often associated with outbreaks [2, 3]. Conjugated polysaccharide vaccines of A, C, W and Y serogroups elicit herd immunity and have suppressed disease worldwide [4]. Because serogroup B polysaccharide mimics human antigens, a vaccine using sub-capsular protein antigens has been licensed for the control of serogroup B disease. The BEXSERO^®^ vaccine (previously known as 4CMenB) (Novartis) incorporates factor H binding protein (fHbp), *Neisseria* adhesin A (NadA), Neisserial Heparin-Binding Antigen (NHBA) and Porin antigen A (PorA) [5].

These sub-capsular antigens undergo antigenic drift to escape natural and vaccine mediated immune selective pressure [6, 7]. To estimate the prevalence of strains expressing the relevant antigen variant and estimate vaccine coverage by BEXSERO^®^, the meningococcal antigen typing system (MATS) is used to measure vaccine elicited antibody binding to strains and hence predict serum bactericidal killing [8, 9]. This approach has revealed that vaccine efficacy varies by jurisdiction from 66–91% globally [10]. Although MATS has been proposed for on-going post-licensure surveillance, it is expensive and time consuming to perform. Increasingly, whole genome sequencing in conjunction with multi-locus sequence typing (MLST) is being used to examine genetic diversity and to predict antigenic diversity over extended time periods [11].

**Fig 1:**
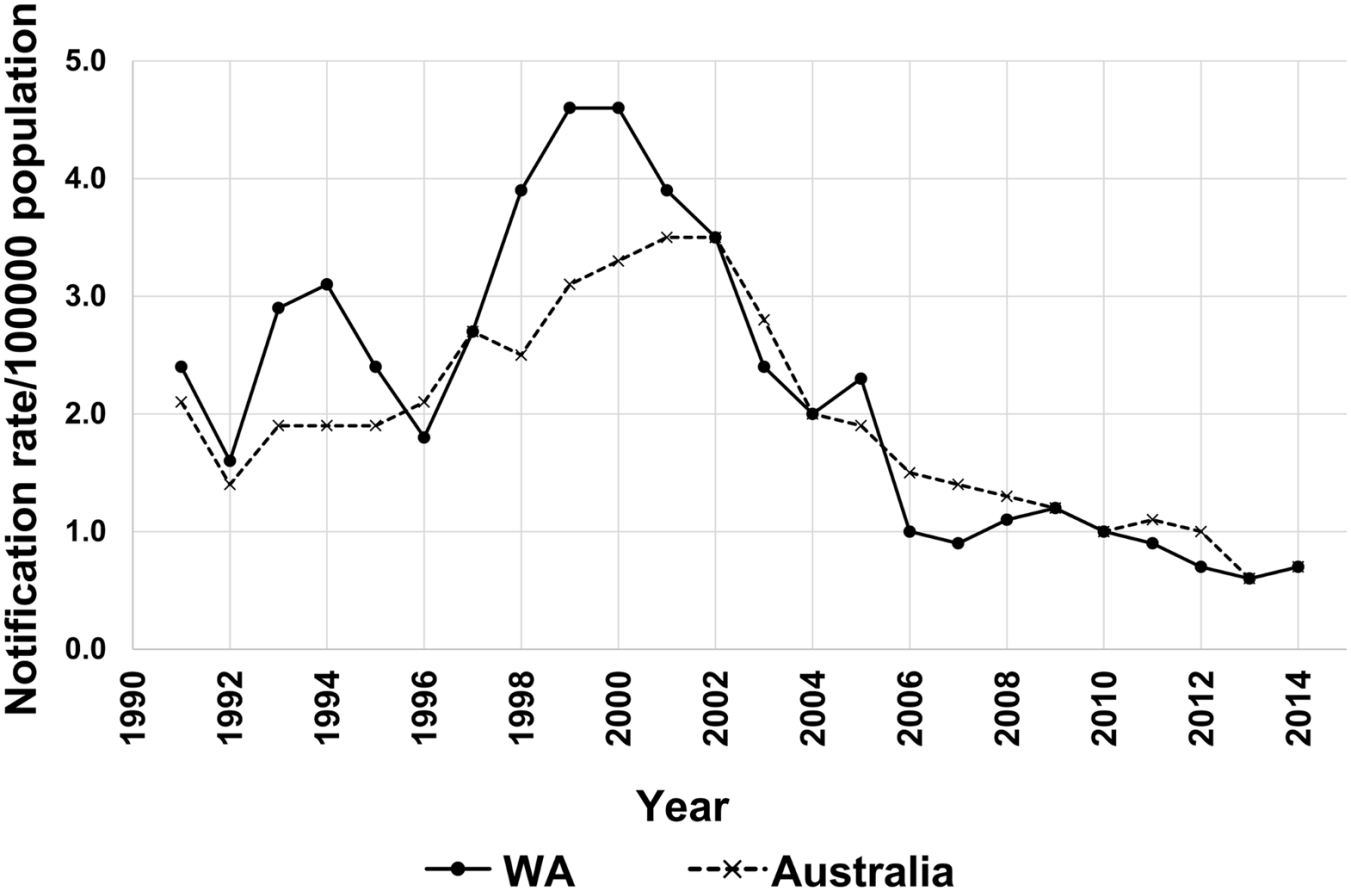
Annual notification rates of meningococcal disease in Australia and Western Australia, 1999–2014. The records represent the National Notifiable Diseases Surveillance System (NNDSS) data for Western Australia and Australia obtained from http://www9.health.gov.au/cda/source/rpt_4.cfm.

In Australia, the majority of the population is located in two coastal regions on either side of the continent—the south-east and east (covering the states of Queensland, New South Wales and Victoria accounting for ~10 million people), and the south-west encompassed within the state of Western Australia (WA). WA is the largest state with a total land area of 2.5 million kilometres and a population of ~2 million inhabitants mostly residing within the capital city of Perth. Although IMD incidence in Australia has ranged from 0.6 to 1.9 per 100,000 population over the past decade, the IMD rate for WA was significantly higher than the mean national IMD rate with hyper-sporadic outbreaks in 1992–1993 (3.1/100,000 population) and 1999–2000 (4.6/100,000 population) (Fig 1). Furthermore, unlike the eastern coastal region where serogroup C meningococci were responsible for the majority of cases, IMD outbreaks in WA were characterised by serogroup B isolates (78% in 2000). As a consequence of the introduction of a national immunisation program for serogroup C disease in 2003, serogroup B disease is currently responsible for the majority of IMD cases across Australia [12].

The current study analysed the temporal relationships of clonal complex and antigenic diversity over 15 years (2000–14) of meningococcal isolates isolated from IMD cases in WA. As far as we are aware, this is the first study to examine the genetic lineage of meningococci circulating in Australia and to assess the diversity of BEXSERO^®^ antigens using whole genome sequencing in addition to MATS.

## Methods

### Bacterial strains and growth conditions

Two hundred and seventy-eight IMD-related meningococci isolated in WA from 2000 to 2014 were collected from the PathWest reference laboratory. Isolates were passaged fewer than 5 times and were cultured under aerobic conditions with 5% CO_2_ at 37°C on GC agar (Oxoid) supplemented with 0.4% glucose, 0.01% glutamine, 0.2 mg/L of cocarboxylase and 5 mg/L of iron(III) nitrate.

### Whole-genome sequencing

Genomic DNA extraction was performed using the DNeasy Blood and Tissue Kit (Qiagen, 69506) and stored as per the manufacturer’s instructions for DNA purification from Gram negative bacteria. Extracted DNA was quantified using Qubit^™^ fluorimetry and 1.0 ng of DNA was used as input to the Illumina Nextera XT library preparation protocol. The genomic DNA was tagmented, indexed by PCR and purified using AMPure XP beads. The “Library Normalization” step was omitted and the library pooling was performed after quantification of the library size using the LabChip^®^ GXII service provided by the Australian Genome Research Facility. The pooled DNA library which contained genomic DNA (12 pM) from 30 meningococcal strains was loaded onto the Miseq and paired-end 250 bp reads were generated.

### Sequence analysis for MLST and antigen profiling

DNA sequences were analysed using the SRST2 program [13] for the detection of the MLST loci [14] and for the genes encoding the antigens found in the BEXSERO^®^ vaccine. Alleles were assigned integers using PubMLST (www.pubmlst.org/neisseria) (accessed on 1^st^ March 2014). The seven housekeeping loci used were internal regions of the *abcZ*, *adk*, *aroE*, *fumC*, *gdh*, *pdhC* and *pgm* genes, each of which had a fixed length of 433 bp, 465 bp, 490 bp, 465 bp, 501 bp, 480 bp, and 450 bp respectively. Molecular typing of fHbp, NHBA, NadA and PorA variants was based on amino acid sequence determination. Nucleotide sequences of the genes detected were imported at the Neisseria Sequence Typing home page to designate the correct variant for each antigen. FHbp was classified into 3 main variants: fHbp-1, -2 and -3. These variants were further classified into subvariants fHbp-1.x, fHbp-2.x and fHbp-3.x (where x denoted the peptide number). The fHbp-2 and -3 variants were further grouped into subfamily A and the fHbp-1 variants were considered as subfamily B. The NadA antigen was grouped into 4 variants: NadA-1, NadA-2/3, NadA-4/5 and NadA-6 [15]. These variants were further classified into the subvariants NadA-1.x, NadA-2/3.x, NadA-4/5.x and NadA-6.x (where x denoted the peptide number). The NHBA antigen was categorised into peptides which were designated NHBA-x (where x denoted the peptide number). As for the PorA antigen, the revised nomenclature scheme was used which consisted of the two major variable regions (VR1 and VR2) and were denoted as P1.VR1,VR2. Every allele detected at the *porA* locus was translated and the PubMLST database was used to determine the VR1 and VR2 variants. At the time of writing, there were 10 VR1 families and 20 VR2 families that contained 262 and 731 unique peptide sequences respectively. The integers of the peptide variants of each of the four antigens (PorA-w: fHbp-x:NadA-y:NHBA-z) was used to generate a BEXSERO^®^ antigen sequence type (BAST) for each isolate. The classification schemes used in this paper and the acronym “BAST” were provided by the PubMLST database hosted by Dr Martin Maiden and Dr Keith Jolley at Oxford University.

### Meningococcal antigen typing system (MATS)

The MATS ELISA assay was performed as previously described by Donnelly et al. [8]. After treating the bacterial suspension with detergents, the bacterial extracts were added to ELISA plates pre-coated with rabbit polyclonal antibodies against fHbp, NadA or NHBA. The plates were incubated at 37°C for 1 h, washed with PBS + 0.05% Tween, incubated with biotinylated rabbit polyclonal antibody at 37°C for 1 h, washed again and incubated with streptavidin-HRP at 37°C for 30 min. Plates were developed with OPD (Sigma) for 20 min at room temperature and the reactions were quenched using 50 μl of 4 N H_2_SO_4_. Plates were read immediately at 492 nm. PorA detection was performed using conventional PCR typing and the presence of a P1.4 subfamily PorA was treated as positive for reactivity.

The MATS ELISA reactivity of each meningococcal isolate was compared to the reactivity of a reference MenB strain for each antigen (strain H44/76 for fHbp, strain 5/99 for NadA and strain NGH-38 for NHBA) by calculating the relative potency (RP) as described by Donnelly et al. [8].

### Statistical Analysis

Categorical variables were examined by the Chi-squared test. GraphPad Prism 5 (Graph PadSoftware Inc., California) was used to perform the analyses. A 5% level of confidence was used and statistical significance was determined with a p value of < 0.05.

Minimal spanning trees were generated using BioNumerics software, version 7 (Applied Maths). All groupings (or clusters) on the minimal spanning trees were computed and assigned by the BioNumerics software. The Cramér’s V coefficient (*V*) was used to measure association of antigenic variants with clonal complexes.

$$V\;=\;\sqrt{\frac{\chi^{2}}{n\left(k-1\right)}}$$

## Results

### Prevalence and Distribution of serogroup and MLST clonal complexes

**Fig 2:**
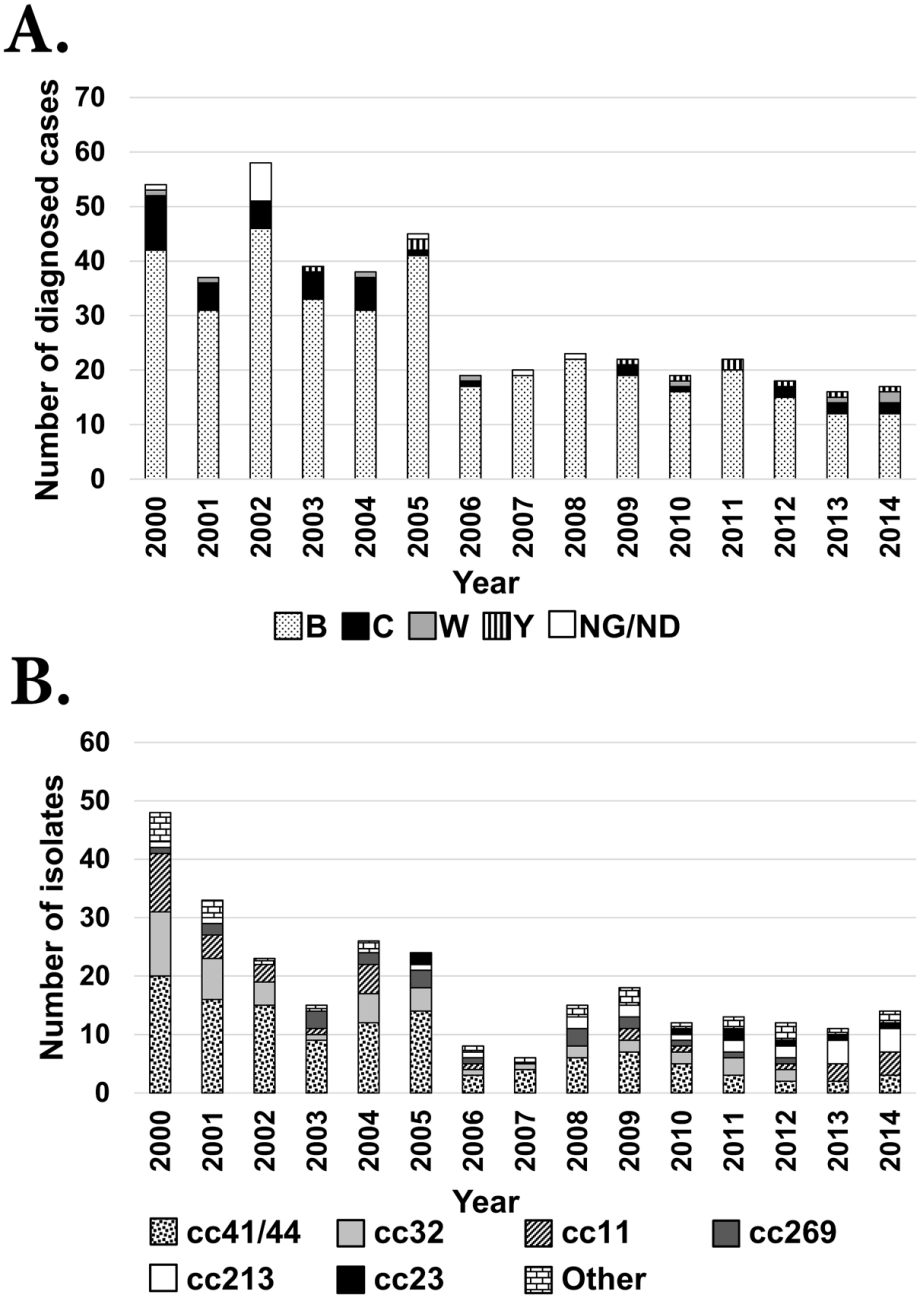
Serogroup (Panel A) and clonal complex (Panel B) distribution per annum from 2000–2014. (A) Serogroup was reported for 436 IMD cases. (B) Clonal complex was reported for 278 cultured isolates. The category “Other” indicates clonal complexes with rare frequency (less than 5 isolates) and represents cc8, cc22, cc35, cc60, cc162, cc167, cc212, cc461, cc1157 and no assigned cc.

Of the 447 notified cases of IMD in WA, the serogroup was determined for 436 isolates. The majority were serogroup B (86%, n = 376) with the remainder being serogroup C (10%, n = 42), serogroup W (2%, n = 8) and serogroup Y (2%, n = 10) (Fig 2A). Sixty-two percent (278/447) of notified IMD cases were cultured and sequenced (S1 and S2 Tables). The proportion of serogroups B, C, W and Y isolates in the sequenced group was 81.7% (n = 227), 12.6% (n = 35), 2.5% (n = 7) and 3.2% (n = 9) respectively.

The most commonly represented clonal complexes were cc41/44 (44%, n = 121), cc32 (16%, n = 45), cc11 (13%, n = 35), cc213 (8%, n = 21) and cc269 (7%, n = 20). The remaining 36 isolates were classified into ten different clonal complexes and contained four isolates belonging to STs not currently assigned to a clonal complex. Only six of the 15 clonal complexes—cc41/44, cc32, cc11, cc269, cc213 and cc23 —persisted for more than six non-consecutive years (Fig 2B).

Clonal complex 41/44 was observed throughout the fifteen years. Of the 121 isolates belonging to cc41/44, 120 were serogroup B and one isolate was a serogroup W (isolated in 2000). Of the thirty STs identified within this cc, ST-146 predominated (36%, n = 44) (S3 Table). Other STs identified in cc41/44 included ST-41 (7%, n = 9), ST-318 (7%, n = 9), ST-154 (7%, n = 8) and ST-42 (6%, n = 7).

Clonal complex 32 which was observed in all years except 2013 and 2014 was predominantly represented by ST-32 (44%, n = 20) and ST-33 (22%, n = 10). All cc32 isolates expressed a serogroup B capsule.

Clonal complex 11 which was predominantly represented by ST-11 (94%, n = 33) was not identified in 2005, 2007, 2008 and 2011. Thirty-one of the cc11 isolates expressed a serogroup C capsule. Of the remaining four cc11 isolates, three isolates expressed a serogroup W capsule and one isolate a serogroup B capsule. Isolates belonging to cc213 and cc269 expressed a serogroup B capsule and were predominantly represented by ST-213 (71%, n = 15) and ST-1214 (20%, n = 4) respectively.

**Fig 3:**
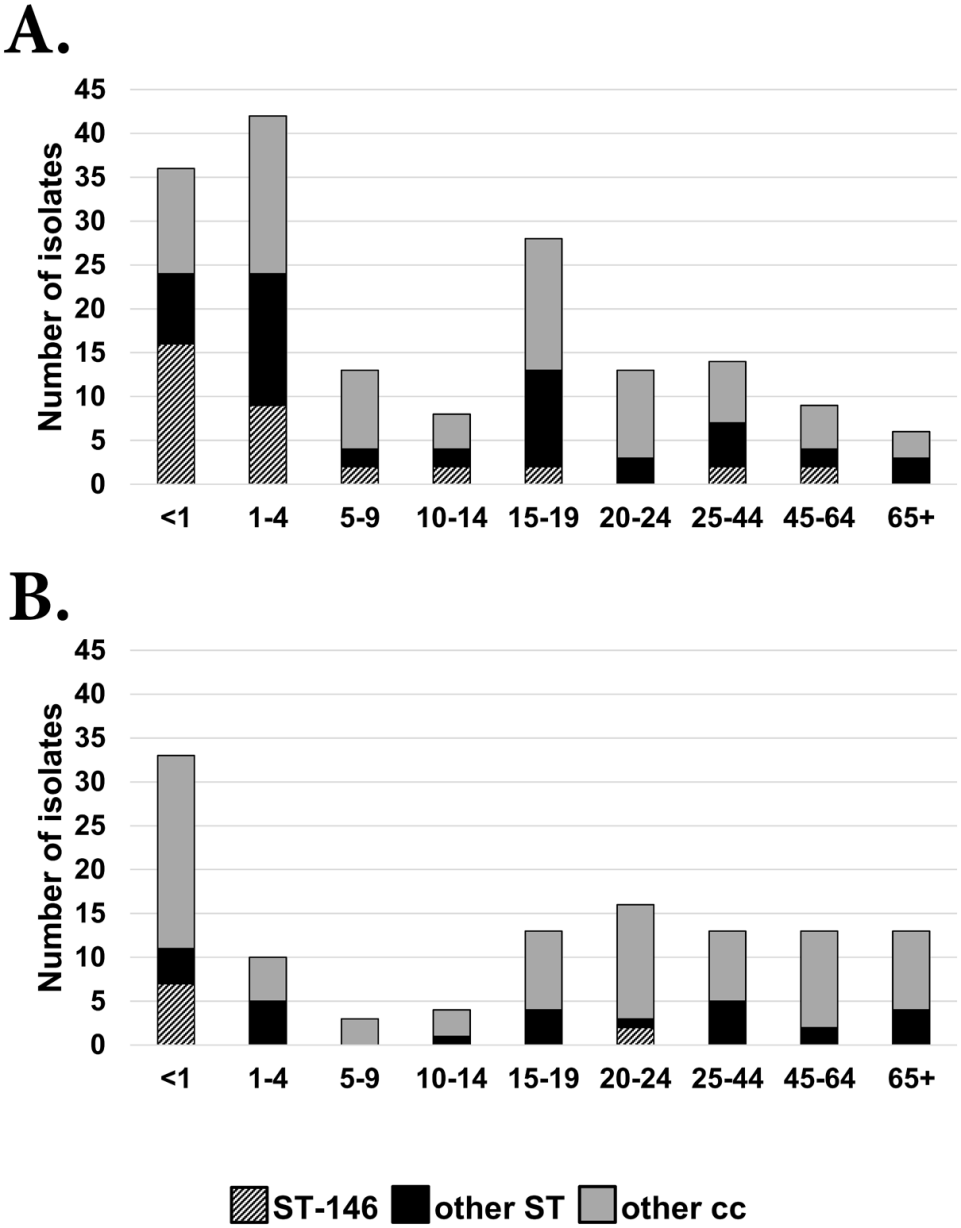
Distribution of cc41/44 in different age groups. Panel A and Panel B show the prevalence of cc41/44 in 2000–05 and 2006–14 respectively. All other clonal complexes other than cc41/44 are shaded in grey, while all cc41/44 isolates are represented by the black and stripped boxes. The stripped boxes represent cc41/44: ST-146 only.

To investigate the hyper-sporadic outbreak in WA and the secondary peak in notification rates that occurred in 2005 (Fig 1), the collection was divided into pre-2006 and post-2005 and analysed by patient age groups (Fig 3). From 2000–05, cc41/44 was the predominant lineage in most age groups, especially in children less than five years of age with a secondary peak in the 15–19 age group. A reduction in the prevalence of cc41/44 was observed post-2005 which occurred concomitantly with the absence of ST-146 in all age groups except for infants and in the 20–24 age group. Therefore, the hyper-sporadic outbreak of IMD from 2000–05 was most likely to be caused by cc41/44 of which ST-146 was the most predominant in that period.

### Diversity and Distribution of PorA

**Fig 4:**
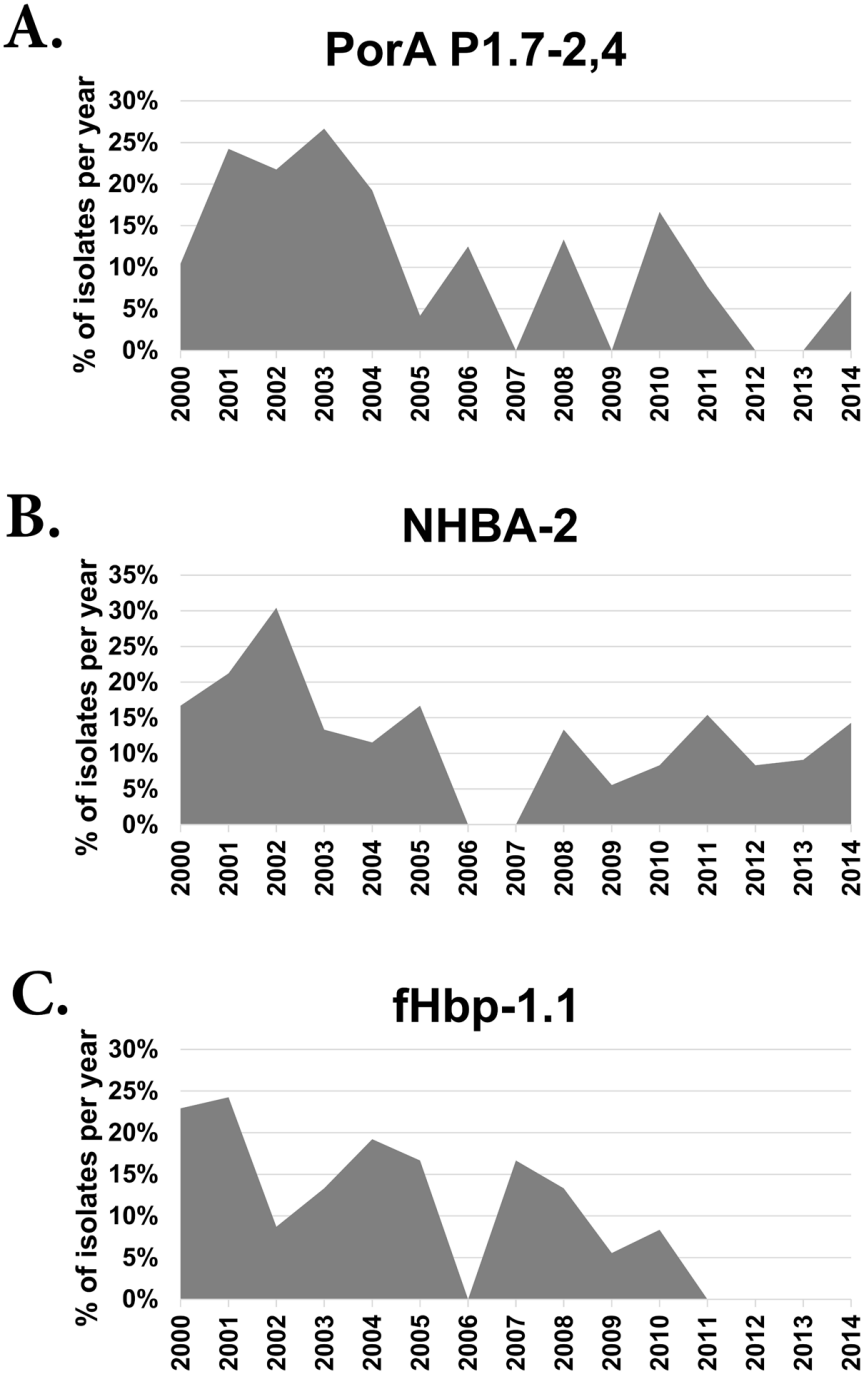
Persistence of the PorA, NHBA and fHbp sub/variants included in BEXSERO^®^ from 2000 to 2014.

A total of 49 PorA subtypes were identified. The most frequently occurring variants were P1.22,14–6 (19%, n = 52), P1.7–2,4 (13%, n = 35) and P1.22,14 (8%, n = 22). None of the porin subtypes were observed in all 15 years consecutively. The P1.7–2,4 subtype which is predicted to be covered by the BEXSERO^®^ vaccine [16] was found in 35 isolates (13%). These isolates predominated pre-2004 (25%) (Fig 4A) and belonged to diverse selection of cc belonging to cc41/44, cc32, cc461 and cc11. All of the major clonal complexes were relatively heterogeneous for the porin type.

### Diversity and Distribution of NHBA

**Fig 5:**
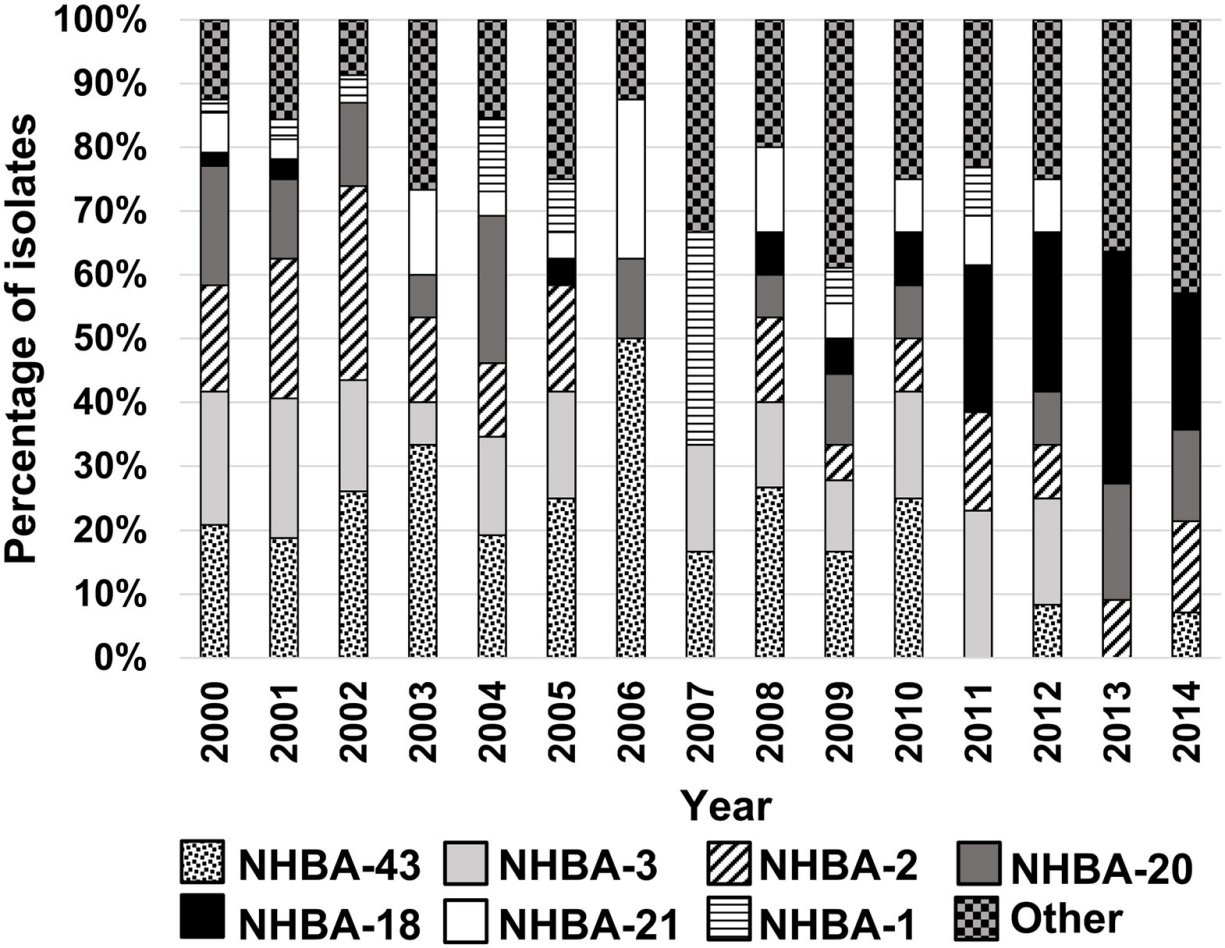
Annual distribution of NHBA peptides from 2000 to 2014. The category “Other” indicates NHBA peptides with rare frequency (less than 7 isolates in total) and represents 28 different NHBA peptides.

The *nhba* gene (NEIS2109) was found in all isolates. Thirty-seven different nucleotide sequences, corresponding to 33 different amino acid sequences, were identified. NHBA-43 (20%, n = 55), NHBA-3 (15%, n = 42), NHBA-2 (15%, n = 41), NHBA-20 (12%, n = 33), NHBA-18 (7%, n = 19), NHBA-21 (4%, n = 12) and NHBA-1 (4%, n = 12) were the most frequent NHBA peptides identified and were observed in 77% (n = 214) of the isolates. The NHBA-2 variant found in the BEXSERO^®^ vaccine, predominated in 2001 (21% of isolates) and 2002 (30% of isolates) (Fig 4B). Temporal replacement of the predominant NHBA antigens was observed over the 15-year period (Fig 5). Whereas NHBA-43 predominated pre-2006, NHBA-18 was the most frequent variant in 2011–14 (Fig 5). While NHBA-43 was most commonly found in isolates from cc11 (n = 30/33, 91%), cc269 (n = 8/12, 67%) and cc41/44 (n = 54/55, 98%), NHBA-18 was most commonly found in cc213 (n = 17/19, 89%) and cc60 (n = 2/19, 11%).

### Diversity and Distribution of NadA

Ninety-one isolates (33%) possessed the *nadA* gene (NEIS1969). The gene was absent in all isolates belonging to cc22, cc23, cc35, cc60, cc162, cc167, cc212, cc269, cc461 and cc41/44. Fourteen isolates (13 from cc32 and one from cc11) encoded a *nadA* allele 19, generating a putative atypical protein (NadA-100 peptide) with a longer anchor domain and an altered distal portion [15]. Eighteen isolates, all belonging to cc11, possessed a *nadA* allele 29 which had the gene interrupted by the insertion sequence IS*1301*, and subsequently would be unable to express an intact NadA peptide. Ten isolates harboured *nadA* variants with internal stop codons and were unlikely to express a peptide (S1 Table). Therefore, 63 (23%) isolates were considered positive for the NadA antigen. NadA-1 and NadA-2/3 variants accounted for 71% (n = 45) and 29% (n = 18) of the positive isolates, respectively, and were predicted to be covered by BEXSERO^®^ vaccine [7, 11].

### Diversity and Distribution of fHbp

The *fHbp* gene sequence (NEIS0349) was present in all isolates and consisted of 58 alleles representing 55 different peptides. Of the nucleotide sequences identified, three alleles (allele 669, allele 743 and allele 746) contained an internal stop codon. Hence, 97% (n = 270) of isolates were considered positive for the expression of the fHbp antigen.

The majority (n = 268) of fHbp positive isolates expressed three variants: fHbp-1 (51%, n = 136), fHbp-2 (36%, n = 97) and fHbp-3 (13%, n = 35). The remaining two isolates, which were both cc11:ST-11:serogroup C isolated in 2000, contained the same allele which encoded an fHbp-1,2,3 hybrid (S1 Fig). Thirty-seven isolates possessed sub-variant fHbp1.1, the fHbp peptide found in the BEXSERO^®^ vaccine. Strains possessing this sub-variant peptide were not identified post-2010 (Fig 4C).

**Fig 6:**
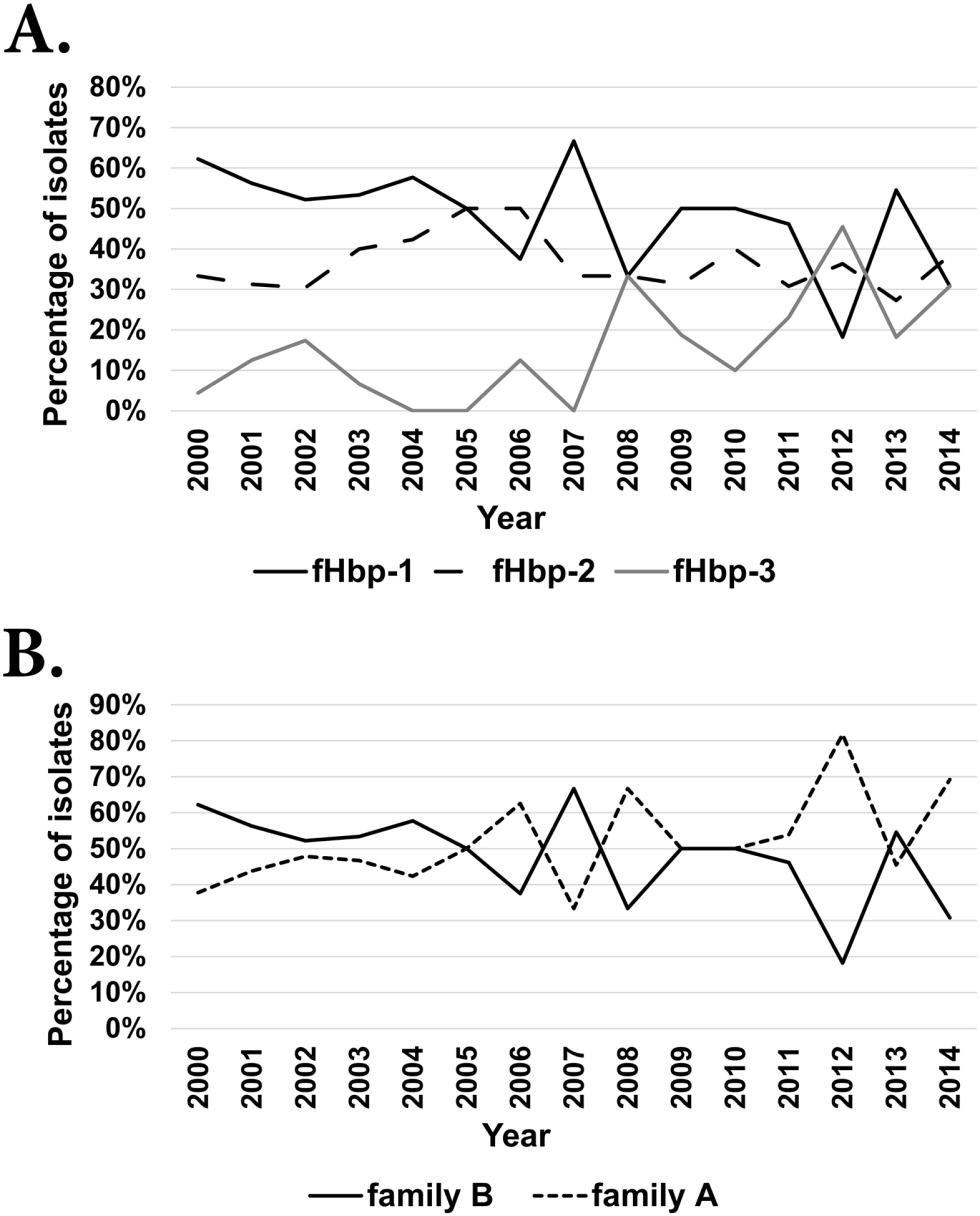
Annual distribution of the fHbp variants based on the Novartis nomenclature (variant 1/2/3, Panel A) and the Pfizer subfamily nomenclature (family A contains variant 1 and family B contains variant 2/3, Panel B).

**Fig 7:**
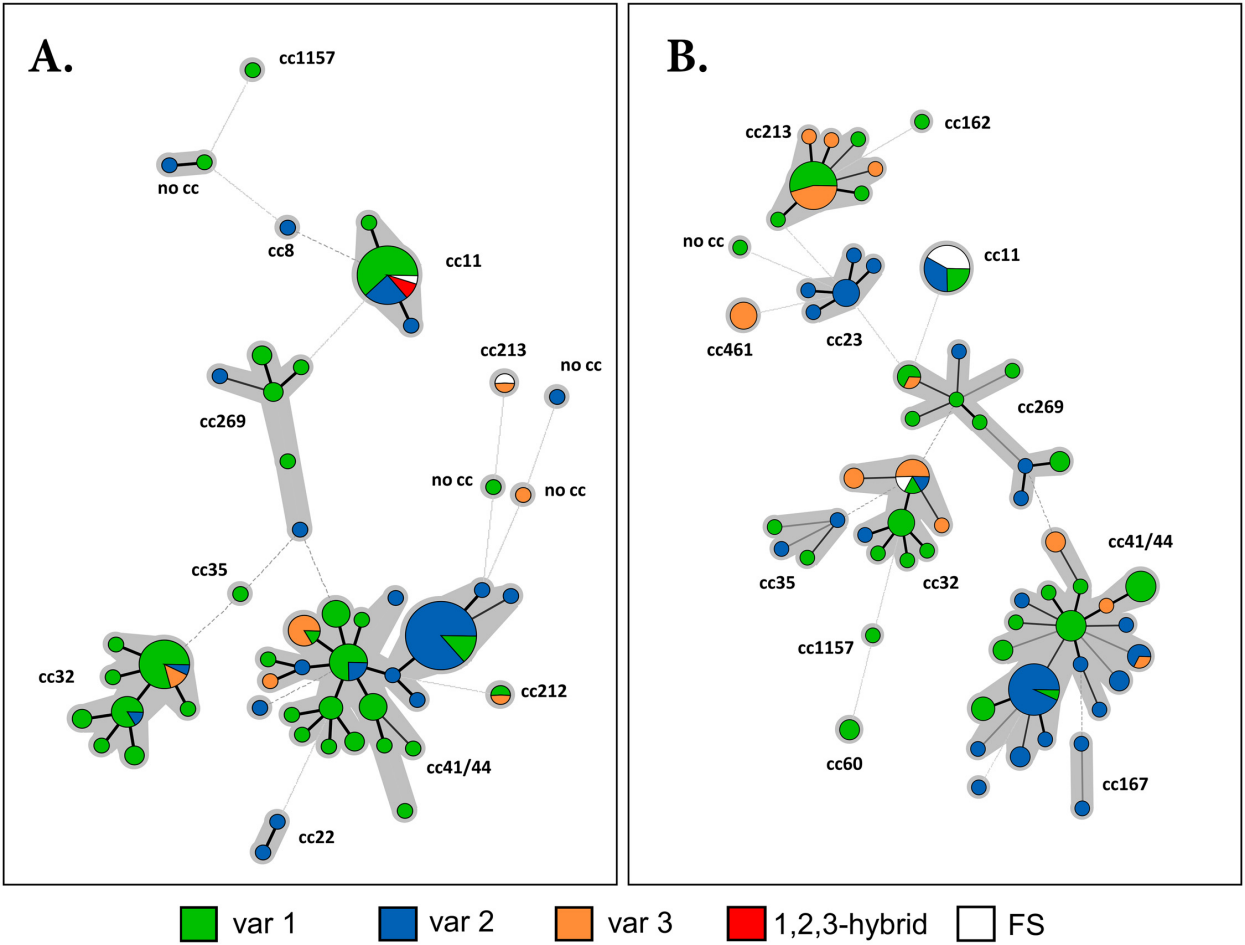
Minimum spanning tree of MLST clonal complexes of *N*. *meningitidis* showing the prevalence of the three fHbp variants during 2000–2004 (Panel A) and 2005–2014 (Panel B). Each circle represents a sequence type (ST) and the size of each circle is proportional to the number of isolates described by the ST. Thick black solid lines denote connections between STs which differ at one locus; grey lines denote connections between STs which differ at two or three loci. Broken lines denote connections between STs that differ at four or more loci. The length of a line connecting two circles is proportional to the number of loci by which the two STs differ. The grey highlight groups circles which are identical at four or more loci and represents STs belonging to one clonal complex were generated by the BioNumerics software. The clonal complexes are indicated outside the grouped circles. The colour of a circle or a sector represents the fHbp variant. FS stands for alleles containing a frameshift. The increase in fHbp-3 was associated with the emergence of cc461, the clonal expansion of cc213 and antigenic drift within cc32.

Overall, isolates possessing fHbp-1 variant predominated during the 15 year period except in 2006 and 2012 when isolates possessing fHbp-2 or fHbp-3 were more prevalent, respectively (Fig 6A). However, when fHbp-2 and fHbp-3 were grouped into Pfizer subfamily A and fHbp-1 into Pfizer subfamily B [17], subfamily B predominated during 2000–04. Post-2004, fHbp-2/3 (subfamily A) was observed to predominate in approximately two year cycles (Fig 6B). To investigate the temporal variance in the prevalence of fHbp variants, a minimum spanning tree of MLST clonal complexes was generated pre-2004 and post-2005 onto which fHbp distribution was mapped (Fig 7). There was no significant change in the prevalence of fHbp-2 in each period. However, there was a significant increase (p = 0.0038) in the proportion of isolates encoding fHbp-3 alleles in cc32 from 2000–04 (2/28, 7%) to 2005–14 (6/17, 35%). In addition, cc213 meningococci possessing fHbp-3 alleles expanded to nineteen isolates post-2004. Lastly, cc461 emerged post-2004 and all four isolates possessed fHbp-3 alleles.

Overall, strains possessing fHbp-3 alleles increased in prevalence while strains possessing fHbp-1 alleles declined in 2005–14. The increase in the prevalence of fHbp-3 during 2005–14 was mostly due to the expansion of strains possessing fHbp-3 alleles in two different clonal complexes and the introduction of a new clonal complex possessing these alleles into the region.

### Diversity and association of BEXSERO^®^ antigen sequence type (BAST) with MLST clonal complexes

The above analysis of the distribution of the individual antigens from the 278 isolates indicates that clonal complexes are quite heterogeneous for some but not all antigens. NHBA association with clonal complex was the strongest (Cramer’s coefficient, V = 0.790) while the association of the other three antigens—fHbp (V = 0.500), NadA (V = 0.649), and PorA (V = 0.468), were less so.

**Fig 8:**
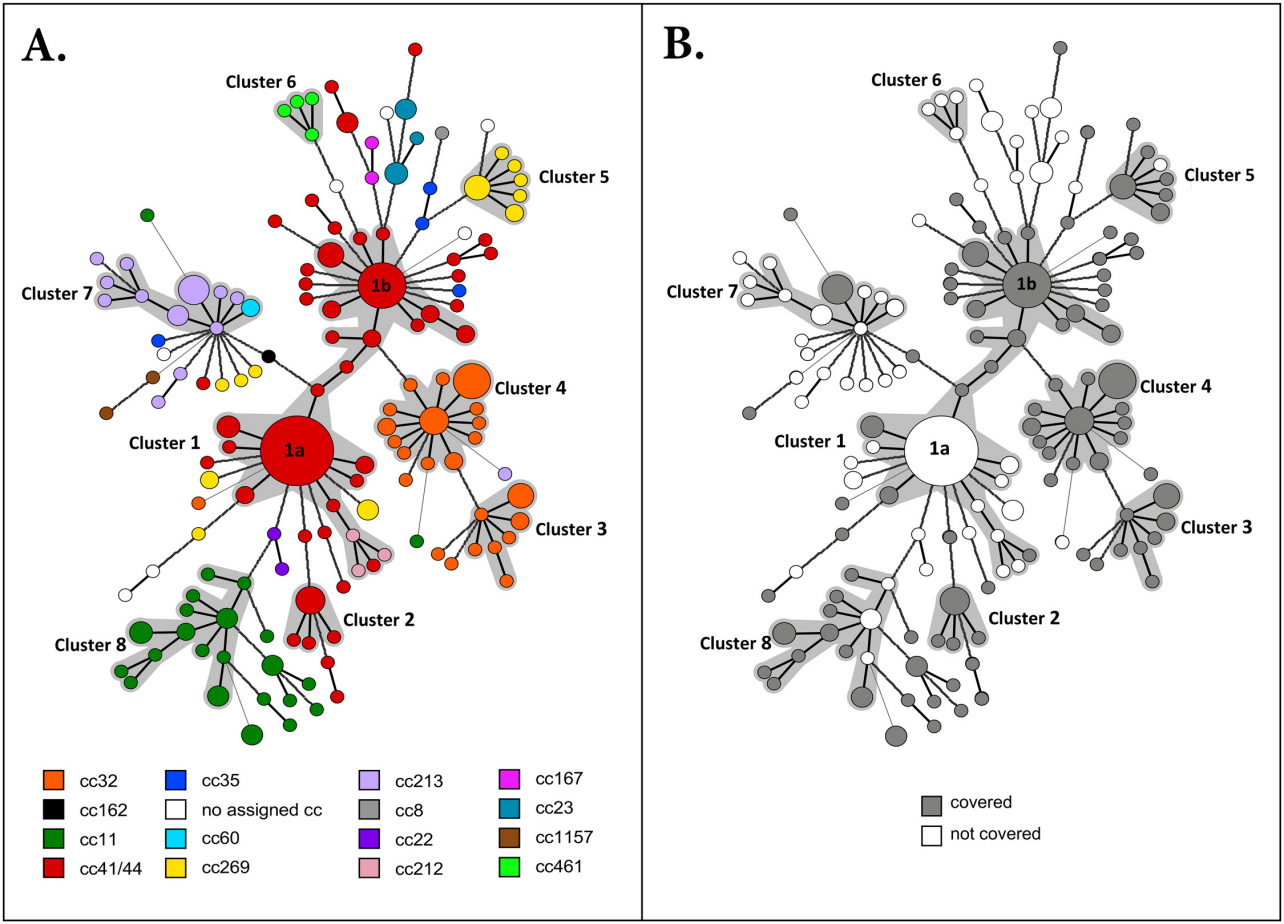
Minimum spanning tree of BEXSERO^®^ antigen sequence type (BAST) of 278 meningococcal isolates using fHbp, NHBA, NadA and PorA peptides. Each circle represents a BAST and the size of each circle is proportional to the frequency at which the BAST was recorded. Thick solid lines connect two circles which represents BASTs that differ at one antigen; broken lines represent two antigenic differences; thin solid lines represent three antigenic differences. Eight main clusters were observed (highlighted in grey) as assigned by the BioNumerics software, each of which contained at least two different BASTs identical at three of the four loci. The most common BAST (labelled 1a) in this study was fHbp-2.19:NadA-absent:NHBA-43:P1.22,14–6. The colours of the circles indicate the MLST clonal complexes of the isolates. A) Different colours show the different clonal complexes in which each BAST was identified. B) BASTs which are predicted to be covered by the BEXSERO^®^ vaccine are showed coloured in grey—these BASTs include one or more of the following antigenic variants: fHbp-1 (except fHbp-1.13), NHBA-1, NHBA-2, NadA-1, NadA-2/3, and P1.7–2,4.

To investigate the diversity and association of the four vaccine antigens (fHbp, NHBA, NadA and PorA) with clonal complex, a minimum spanning tree was generated based on the antigenic variants (Fig 8A). Overall, there were 55 fHbp peptides, 16 NadA peptides, 34 NHBA peptides and 49 PorA subtypes. Random integers assigned to each PorA subtype and the peptide numbers for fHbp, NadA and NHBA were used to describe each isolate by a combination of four integers which represented a **B**EXSERO^®^ **a**ntigen **s**equence **t**ype (BAST) (see methods). A total of 152 BASTs were present in this dataset and were grouped into eight main clusters based on the conservation of the four antigen variants (Table 1). The defined clusters contained 78 BASTs and each cluster contained at least four BASTs which differed by only one antigen. Approximately 70% (n = 194/278) of the isolates fell into the eight clusters, with 87% (n = 168/194) belonging to the hyper-invasive lineages cc41/44, cc32, cc11 and cc269. The remaining 30% (n = 84/278) of strains were characterised by a total of 74 BASTs which differed from one another at two or more of the four loci. In this subset of the collection 37% (n = 31/84) of strains belonged to rare clonal complexes not associated with the hyper-virulent lineages while 63% (n = 53/84) were associated with hypervirulent lineages.

**Table 1:** BEXSERO^®^ antigen sequence type diversity within WA meningococcal population.

	NHBA peptide	fHbp subvariant	NadA subvariant	PorA subtype	Clonal complex	No. of isolates
**Cluster 1a**	NHBA-43	fHbp-1.1	-	P1.7–2,4	cc41/44	1
**(n = 58)**	NHBA-43	fHbp-1.1	-	P1.22,14–6	cc41/44	1
	NHBA-43	fHbp-1.4	-	P1.22,14–6	cc41/44	4
	NHBA-43	fHbp-1.8	-	P1.22,14–6	cc41/44	2
	NHBA-43	fHbp-2.118	-	P1.22,14–6	cc41/44	1
	NHBA-43	fHbp-2.16	-	P1.22,14–6	cc41/44	2
	NHBA-43	fHbp-2.19	-	P1.22,14–6	cc41/44	41
	NHBA-43	fHbp-2.19	-	P1.22,13–9	cc41/44	1
	NHBA-43	fHbp-2.19	-	P1.19–3,15	cc41/44	1
	NHBA-47	fHbp-2.19	-	P1.19,15	cc41/44	1
	NHBA-47	fHbp-2.19	-	P1.19–3,15	cc212	1
	NHBA-47	fHbp-1.697	-	P1.19–3,15	cc212	1
	NHBA-47	fHbp-3.31	-	P1.19–3,15	cc212	1
**Cluster 1b**	NHBA-214	fHbp-1.1	-	P1.7–2,4	cc41/44	1
**(n = 34)**	NHBA-2	fHbp-2.16	-	P1.7–2,4	cc41/44	2
	NHBA-2	fHbp-3.111	-	P1.7–2,4	cc41/44	5
	NHBA-2	fHbp-1.1	-	P1.7–2,4	cc41/44	2
	NHBA-2	fHbp-1.4	-	P1.7–2,4	cc41/44	17
	NHBA-2	fHbp-1.4	-	P1.7–2,4–13	cc41/44	2
	NHBA-2	fHbp-3.30		P1.7–2,4–13	cc41/44	2
	NHBA-2	fHbp-1.4	-	P1.18–1,3	cc41/44	1
	NHBA-2	fHbp-1.4	-	P1.19-,15	cc41/44	1
	NHBA-2	fHbp-1.4	-	P1.5–1,10–4	cc41/44	1
**Cluster 2**	NHBA-1	fHbp-1.14	-	P1.5–2,10	cc41/44	1
**(n = 9)**	NHBA-1	fHbp-1.14	-	P1.5–2,10–11	cc41/44	6
	NHBA-1	fHbp-1.89	-	P1.5–2,10–11	cc41/44	1
	NHBA-1	fHbp-1.novel	-	P1.5–2,10–11	cc41/44	1
**Cluster 3**	NHBA-3	fHbp-1.69	NadA-1.100	P1.7,16–26	cc32	1
**(n = 12)**	NHBA-3	fHbp-2.21	NadA-1.100	P1.7,16–26	cc32	2
	NHBA-3	FS	NadA-1.100	P1.7,16–26	cc32	1
	NHBA-3	fHbp-3.31	NadA-1.100	P1.7,16–26	cc32	5
	NHBA-3	fHbp-3.29	NadA-1.100	P1.7,16–26	cc32	1
	NHBA-3	fHbp-3.29	NadA-1.100	P1.7–2,16–26	cc32	1
	NHBA-3	fHbp-3.novel	NadA-1.100	P1.7–2,16–26	cc32	1
**Cluster 4**	NHBA-3	fHbp-2.16	NadA-1.1	P1.19,15	cc32	1
**(n = 27)**	NHBA-3	fHbp-1.1	-	P1.19,15	cc32	1
	NHBA-3	fHbp-1.1	NadA-1.1	P1.19,15	cc32	6
	NHBA-3	fHbp-1.1	NadA-1.1	P1.19–1,26	cc32	1
	NHBA-3	fHbp-1.1	NadA-1.1	P1.22–1,14	cc32	1
	NHBA-3	fHbp-1.1	NadA-1.1	P1.5–1,10–4	cc32	1
	NHBA-3	fHbp-1.1	NadA-1.1	P1.7,16	cc32	10
	NHBA-3	fHbp-1.1	NadA-1.1	P1.7,16–26	cc32	2
	NHBA-3	fHbp-1.1	NadA-1.1	P1.7,30–3	cc32	1
	NHBA-3	fHbp-1.1	NadA-1.1	P1.7,30–7	cc32	2
	NHBA-3	fHbp-1.1	NadA-1.1	P1.7–11,30–8	cc32	1
**Cluster 5**	NHBA-21	fHbp-1.15	-	P1.5–2,10–2	cc269	1
**(n = 10)**	NHBA-21	fHbp-1.15	-	P1.7,30–7	cc269	1
	NHBA-21	fHbp-1.15	-	P1.7–2,16	cc269	1
	NHBA-21	fHbp-1.15	-	P1.19–1,15–11	cc269	4
	NHBA-21	fHbp-1.622	-	P1.19–1,15–11	cc269	2
	NHBA-21	fHbp-3.novel	-	P1.19–1,15–11	cc269	1
**Cluster 6**	NHBA-598	fHbp-3.47	-	P1.19–2,13–1	cc461	1
**(n = 4)**	NHBA-118	fHbp-3.47	-	P1.19–2,13–1	cc461	1
	NHBA-118	fHbp-3.47	-	P1.7–11,30–8	cc461	1
	NHBA-118	fHbp-3.47	-	P1.7–1,4–1	cc461	1
**Cluster 7**	NHBA-18	fHbp-1.13	-	P1.5,2	cc60	2
**(n = 19)**	NHBA-18	fHbp-1.430	-	P1.22,14	cc213	7
	NHBA-18	fHbp-1.13	-	P1.22,14	cc213	1
	NHBA-18	fHbp-1.13	(allele 34)	P1.22,14	cc213	1
	NHBA-18	FS	(allele 34)	P1.22,14	cc213	1
	NHBA-18	fHbp-3.45	(allele 34)	P1.18,25	cc213	1
	NHBA-18	fHbp-3.45	(allele 34)	P1.22,14	cc213	1
	novel	fHbp-3.45	(allele 34)	P1.22,14	cc213	1
	NHBA-18	fHbp-3.45	-	P1.22,14	cc213	3
	NHBA-18	fHbp-3.30	-	P1.22,14	cc213	1
**Cluster 8**	NHBA-20	FS	(allele 29)	P1.18–1,3	cc11	1
**(n = 21)**	NHBA-20	fHbp-1.1	(allele 29)	P1.18–1,3	cc11	1
	NHBA-20	fHbp-1.37	(allele 29)	P1.5,2	cc11	3
	NHBA-20	FS	(allele 29)	P1.5,2	cc11	1
	NHBA-20	fHbp-1.1	(allele 29)	P1.5–1,10–1	cc11	1
	NHBA-20	fHbp-1.11	(allele 29)	P1.5–1,10–1	cc11	1
	NHBA-20	fHbp-1.11	NadA-2/3.2	P1.5–1,10–1	cc11	1
	NHBA-20	fHbp-1.11	NadA-2/3.2	P1.5–1,10–8	cc11	4
	NHBA-20	FS	NadA-2/3.2	P1.5–1,10–8	cc11	1
	NHBA-20	FS	(allele 29)	P1.5–1,10–8	cc11	3
	NHBA-20	fHbp-1.10	(allele 29)	P1.5–1,10–8	cc11	1
	NHBA-20	fHbp-1.11	(allele 29)	P1.5–1,10–8	cc11	2
	NHBA-20	fHbp-1.601	(allele 29)	P1.5–1,10–8	cc11	1

Each cluster was observed to contain one major NHBA peptide variant with diversification mainly driven by the presence of fHbp and PorA variants or the presence/absence of NadA. When overlaid with genetic lineage, the BAST clusters were found to be highly associated with clonal complex. Cluster 1 and 2 represented 81% (n = 98/121) of cc41/44 isolates. Cluster 1a was characterised by NHBA-43 and NHBA-47 associated with 8 different fHbp variants and 5 different PorA subtypes. This cluster also contained the predominant BAST—fHbp2.19:NadA-absent:NHBA-45:P1.22,14–6. Cluster 1b was characterised by NHBA-2 and NHBA-214 associated with 5 different fHbp variants and 5 different PorA subtypes. Cluster 2 contained four BASTs, all of which were identical for NHBA and showed diversification with 3 fHbp variants and 2 PorA subtypes. Cluster 3 and 4 contained 87% (n = 39/45) of all cc32 isolates in the collection and were characterised by the possession of NHBA-3 and two NadA variants (NadA1.100 in cluster 3 and NadA1.1 in cluster 4). Cluster 5 contained 50% (n = 10/20) of all cc269 isolates all of which possessed NHBA-21. Cluster 6 contained all four isolates of cc461 which possessed a common NHBA-118 variant and fHbp-3.47 variant but diverse PorA subtypes. Cluster 7 represented 81% (n = 17/21) and 100% (n = 2/2) of cc213 and cc60 isolates respectively. In this case, both the NHBA peptide and PorA subtype remained conserved while five different fHbp peptides were found. Lastly, cluster 8 contained 60% (n = 21/35) of cc11 and was founded on the conserved possession of the NHBA-20 peptide. This cluster did not contain a predominant BAST due to the high diversity of fHbp variants and PorA subtypes. When the criterion for cluster formation was relaxed to two common variants, 89% (n = 31/35) of cc11 was included.

In conclusion, the BAST minimal spanning tree revealed that there is an association of BAST and antigen combination with clonal complex which was most apparent in the hyper-virulent lineages in this limited dataset over the 15 year time scale.

### Estimating Vaccine Coverage using WGS and MATS Assay

BAST was then used to estimate the coverage of the BEXSERO^®^ vaccine in WA. A serogroup B meningococcal isolate was predicted to be covered by the BEXSERO^®^ vaccine induced serum antibody response if the genome harboured intact genes for one or more of the following antigenic variants—fHbp-1, NadA-1 or NadA-2/3, NHBA-2, P1.4 subfamily PorA. This approach predicted that 63% of serogroup B isolates (n = 143/227) represented by 94 BASTs, exhibited an annual range of coverage of between 44% to 91% (S2 Fig). However, these predictions did not take into account the expression of the antigens. Consequently, MATS ELISA assay [8] was performed on 43 serogroup B isolates which represented 77% (n = 43/56) of isolates from the 2007–2011 time period and possessed 31 different BASTs (S4 Table). All isolates possessing either NHBA-1 or NHBA-2 had relative potency (RP) values consistent with positive antibody binding to the strain. As expected, isolates harbouring the fHbp variant 2 or 3 were negative for antibody binding. Of the isolates harbouring fHbp-1 variants, those possessing the fHbp-1.13 subvariant (allele 13) were also negative for MATS. Lastly, all nine isolates which possessed NadA-1 had an RP value below the lower limit of quantitation [8]. These isolates were scored as being positive since Fagnocchi et al. [18] had previously shown that *in vitro* grown strains with MATs RP below the positive bactericidal threshold for NadA either through repression or phase variation expressed higher levels of NadA in an infected rat model and to levels sufficient for bactericidal killing by vaccinated sera.

The parameters for predicting vaccine coverage were re-calculated based on the MATS data which led to the exclusion of fHbp-1.13 and the inclusion of NHBA-1. The combined BAST and MATS analysis estimated the vaccine coverage for the serogroup B isolates was 60% (n = 137/227) and fluctuated annually between 40% and 82% (S2 Fig). The inclusion of isolates expressing other serogroups did not substantially alter the predicted vaccine coverage which was estimated to be 60% (n = 168/278) with an annual range of 33% to 78% (S2 Fig).

Due to BAST diversity, the predicted coverage for the BEXSERO^®^ vaccine varied for the different lineages in the collection. Of the BASTs identified in the hyper-invasive lineages individually, 15% (n = 2/13), 50% (6/12), 73% (n = 33/45) and 83% (n = 19/23) were predicted to be covered for cc213, cc269, cc41/44 and cc11 respectively. Unlike other lineages, all BASTs found in cc32 isolates (n = 24/24) were predicted to be covered by the vaccine (Fig 8B) because in this lineage, all isolates harboured a *nadA* gene which encoded a NadA-1 variant or the fHbp1.1 subvariant. The most common BAST in this collection was represented by forty-one isolates belonging to cc41/44, of which 85% (n = 35/41) were ST-146, and was not predicted to be covered by the vaccine by MATS and BAST.

## Discussion

Clonal complex 41/44 was the most common genetic lineage in WA over the 15-year period but reduced in prevalence post-2010. Prior to 2010, cc41/44, mainly represented by ST-41 and ST-44, was a frequent cause of serogroup B outbreaks in many countries [11, 19–25]. In WA, however, cc41/44:ST-146 was the predominant cause of IMD in children <5 years during the hyper-endemic IMD period between 2000 and 2005 and accounted for 52% of all cc41/44 cases of disease. The antigenic profile of the ST-146 lineage is also consistent with the most prevalent strain causing IMD in the aboriginal population during 2000–2011 identified by Boan et al. [26]. The parallel decline in IMD cases and in cc41/44 incidence observed during the studied time-frame suggests that the hyper-sporadic outbreak during 1995–2005 (Fig 1) was potentially caused by cc41/44 isolates, of which ST-146 was the major sequence type. Globally, ST-146 has been rarely recorded outside WA (PubMLST database) and has not been reported as a causative agent of an IMD outbreak in those regions.

Association of surface antigen variants of FetA, PorA and PorB with genetic lineages of meningococci was first described by Urwin et al. [27] and further extrapolated by Watkins and Maiden [28] who reported that each geographic region is characterised by a meningococcal population of limited strain types exhibiting non-overlapping PorA:FetA profiles which were relatively stable over a 74-year time period. Bambini et al. [11] sampled 165 isolates over a 50-year time frame from the Netherlands and confirmed that there was antigenic structuring of the BEXSERO^®^ vaccine antigens. Our study has extended these observations to include 278 isolates from Australia which were shown to be characterised by 152 BASTs. In this collection each BAST was restricted to only one cc. Despite no vaccine selection pressure, different BASTs were observed within the same cc which may indicate ongoing mutation and antigenic diversification due to natural immunity elicited by carriage [27, 28]. It is interesting to note that despite the relative isolation of Western Australia from other population centers, the strain diversity within this collection is similar to that observed in previous studies and very few isolates were unassigned an ST or possessed a novel antigen allele.

Nissen et al. [29] had previously used the MATS ELISA assay to predict an average national vaccine coverage of 76% for Australia using 373 invasive serogroup B strains isolated during 2007–2011. Since this previous study contained only 52 isolates from WA, BAST profiling was used to compute the putative vaccine coverage of BEXSERO^®^ in this region in the last 15 years. The MATS ELISA performed in this study assessed 32 individual antigenic variants of fHbp, NHBA and NadA which represented 66% (31/47) of the BASTs identified during the 2007–2011 time period. Although variants of fHbp-1 are generally considered to be covered by the vaccine response [8], all three isolates possessing the fHbp-1.13 variant had RP values below the lower limit of quantitation and were predicted to not be covered by the vaccine. In addition to the five isolates encoding the NHBA-2 variant incorporated in the BEXSERO^®^ vaccine, all four isolates possessing the NHBA-1 variant were covered by MATS due to this antigen (S4 Table). Using these observations, a meningococcal isolate was predicted to be covered by the BEXSERO^®^ vaccine if the BAST profile contained one or more of the following variants—fHbp-1 (except fHbp-1.13), NHBA-1, NHBA-2, NadA-1, NadA-2/3 and P1.4 subfamily PorA.

In the period 2007–2011, the predicted vaccine coverage by MATS was 58% (25/43) and was in general agreement with the average predicted coverage of 55% (31/56) obtained using BAST profiling on all serogroup B strains isolated during the same time period. Considering that both techniques for estimating vaccine coverage were in general agreement, extrapolation across the 15 year period resulted in an predicted coverage of 60% using BAST profiling on serogroup B isolates with an annual range of 40% to 82%. The lower average coverage for WA compared to the national coverage was mostly attributable to the high prevalence of cc41/44:ST-146 until 2005. The majority of these strains possessed a BAST (fHbp-2.19:NadA-absent:NHBA-43:P1.22,14–6) that MATS confirmed, using six isolates, was not covered by the BEXSERO^®^ immune response (S4 Table). Periods of low vaccine coverage in years post-2005 were not a result of the resurgence of cc41/44:ST-146 but were characterised by increased diversity of clonal complexes expressing BASTs which were not predicted to be covered by the vaccine. However, MATS or serum bactericidal assays would need to be performed on these strains to validate this observation since these assays provide a measurement of antigen expression which is not assessed using BAST profiling alone. Frosi et al [30] have previously shown that MATS underestimates vaccine coverage due to the effect of bactericidal antibodies raised against minor antigens in the vaccine which are not examined in the MATS assay but contribute to killing in serum bactericidal assays.

The lower predicted MATS coverage in WA when compared to the mean average for Australia may be a result of genetic differentiation of isolates between the eastern and western seaboards of Australia. Previous studies of MATS coverage have reported average coverage within one country or several countries [10]. However, meningococcal populations have been shown to be genetically differentiated between countries across Europe, such as the comparison of Norway, Greece and the Czech Republic by Yazdankhah et al. [19]. While Norway and Greece are separated by approximately 2400 km, Australia has an estimated land area of ~8.6 million km^2^ with a width of ~4100 km with the majority of the inhabitants situated on either side of the continent. The observation that outbreaks of serogroup B disease predominated in Western Australia between 1999 to 2005 while the eastern seaboard experienced elevated serogroup C disease, may support the hypothesis that the population structure of circulating meningococci may be different for the eastern and western seaboards of Australia.

Currently, the IMD rates in WA are at a historical low of 0.9/100,000 population. Serogroup B predominates in children <5 years while increasingly serogroup Y and W are contributing to IMD in the elderly and young adolescent population, respectively. The elevated levels of IMD by serogroup W isolates is the result of cc11-ST11-MenW, a hyper-virulent lineage causing outbreaks in England and Wales [31], Argentina [32], Brazil [33] and Chile [34]. This pattern of IMD incidence is similar to that seen in the UK which has recently introduced a schedule of BEXSERO^®^ vaccination in <5 years old age group and a campaign to vaccinate the adolescent population with conjugate A/C/Y/W vaccine. A similar scheme should be considered for WA based upon our observations. However, with the presence of endemic strains such as cc41/44:ST-146 and a high proportion of strains from diverse cc both of which are predicted to be not covered by BEXSERO^®^ using either BAST or MATS assays, continued surveillance is desirable to determine whether the widespread use of the vaccine in this region would result in strain replacement.

## Supporting Information

S1 Fig Maximum likelihood tree of fHbp peptides (500 bootstraps) showing the clustering of fHbp-1 (green), fHbp-2 (blue), fHbp-3 (orange) and fHbp-1,2,3 hybrid (red). The red asterisk shows the position of the peptide encoded by the fHbp-1,2,3 hybrid allele identified in the WA collection. The phylogenetic tree was generated using MEGA6 and edited using FigTree v1.4.2.
(TIF)

S2 Fig Estimated annual coverage of the BEXSERO^®^ vaccine for serogroup B (Panel A) and all serogroups (Panel B) in Western Australia from 2000 to 2014. The number of BASTs estimated to be covered by the vaccine annually is shown in Panel C.
(TIF)

S1 Table Number of cases and number of recovered isolates used for sequencing. (DOCX)

S2 Table List of meningococcal isolates included in this study including clonal complex and BESERO^®^ antigen typing data. (DOCX)

S3 Table Genetic diversity of *N*. *meningitidis* isolates in this study. (DOCX)

S4 Table Data for the MATS ELISA assay performed in this study. (DOCX)

## Abbreviations

ST: sequence type
cc: clonal complex
MLST: multi-locus sequence typing
BAST: BEXSERO^®^ antigen sequence type
MATS: meningococcal antigen typing system
fHbp: factor H binding protein
NHBA: neisserial heparin binding antigen
NadA: *Neisseria* adhesion A
PorA: porin A
SRST2: short read sequence typing
IMD: invasive meningococcal disease
WA: Western Australia
