# Supplementary figures and images for: Temporal Changes in BEXSERO® Antigen Sequence Type Associated with Genetic Lineages of Neisseria meningitidis over a 15-Year Period in Western Australia
Source: PLoS One. 2016 Jun 29;11(6):e0158315. doi: 10.1371/journal.pone.0158315 (PMC4927168; doi:10.1371/journal.pone.0158315)

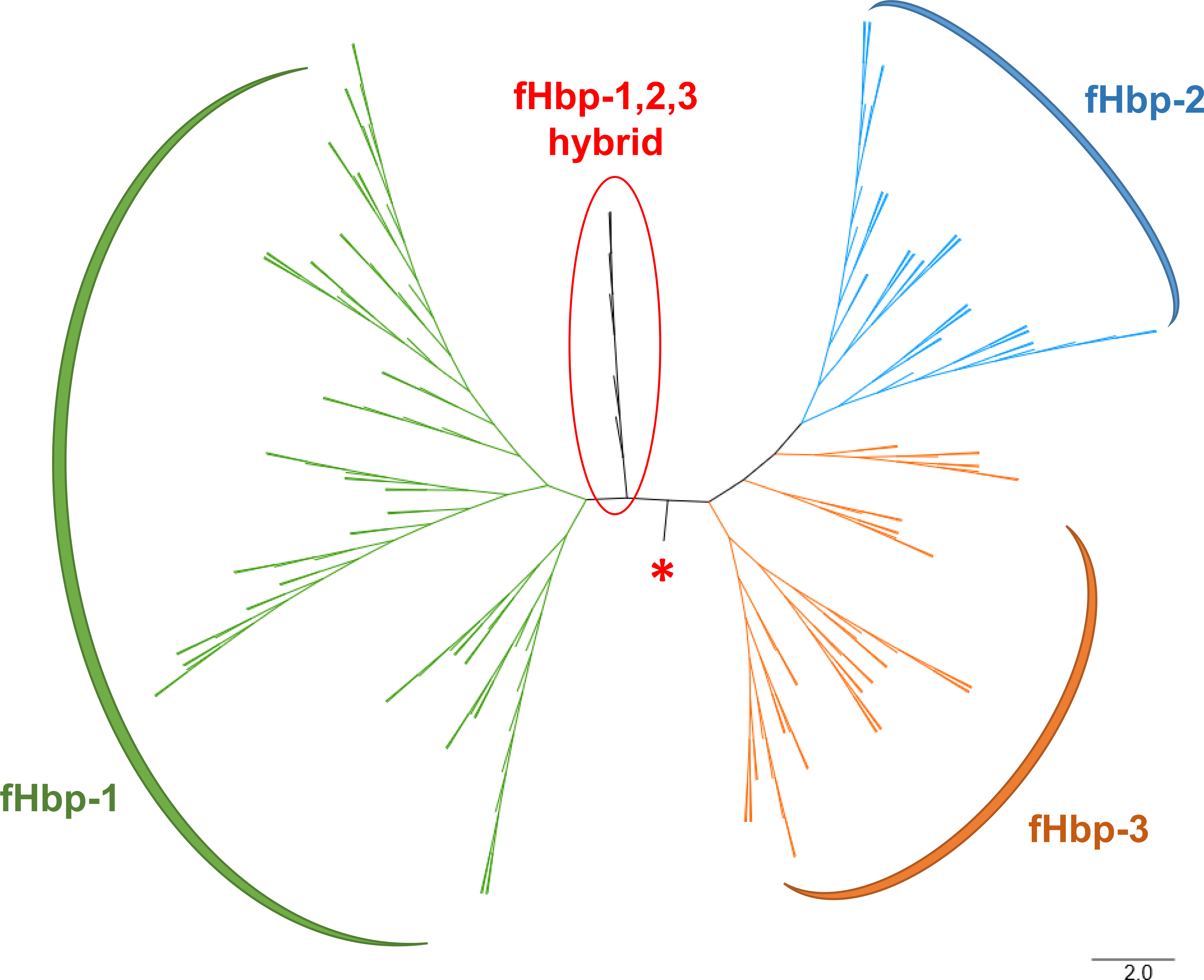

Supplement: S1 Fig — The red asterisk shows the position of the peptide encoded by the fHbp-1,2,3 hybrid allele identified in the WA collection. The phylogenetic tree was generated using MEGA6 and edited using FigTree v1.4.2. (TIF) [file pone.0158315.s001.tif]

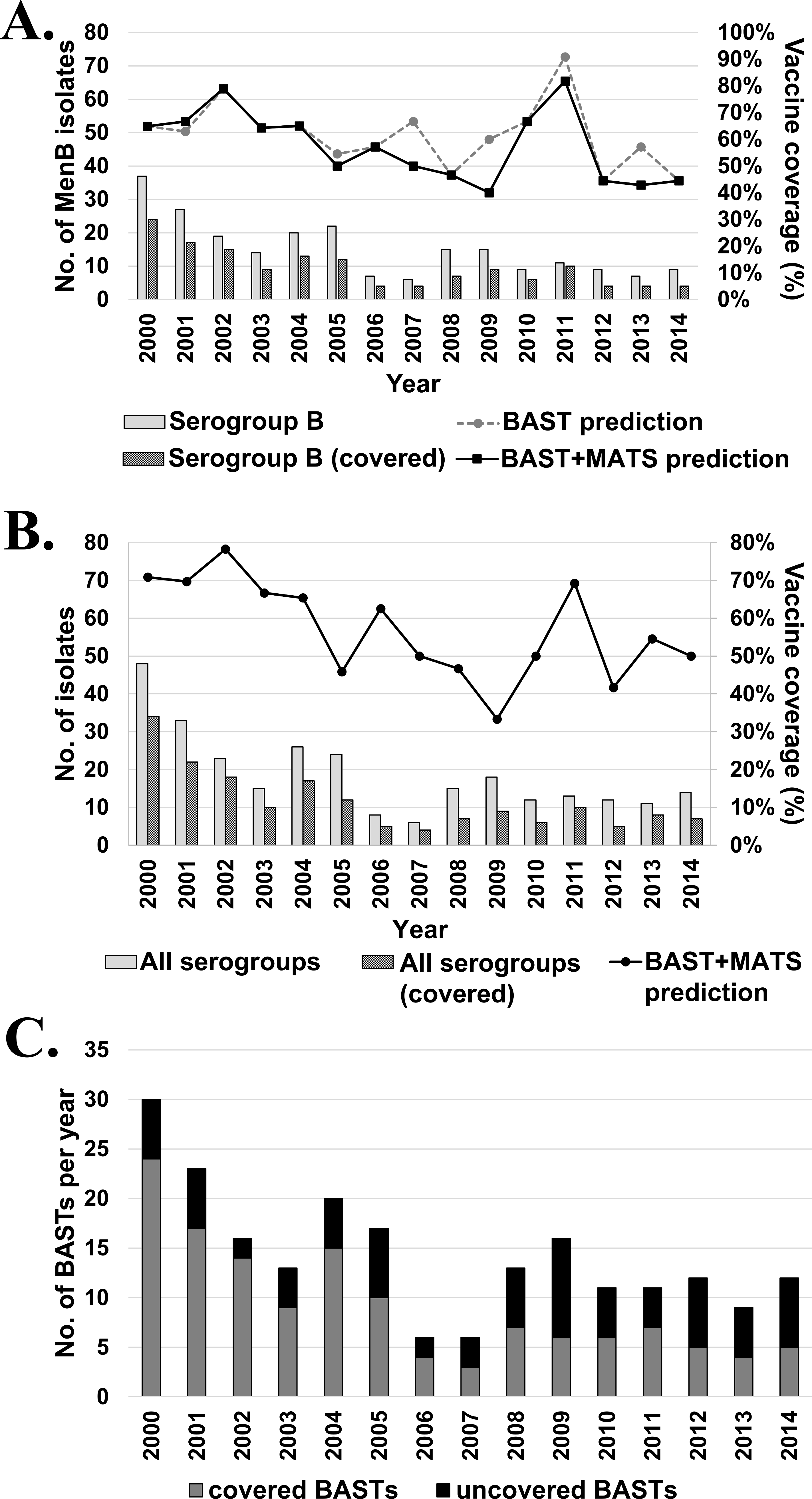

Supplement: S2 Fig — The number of BASTs estimated to be covered by the vaccine annually is shown in Panel C. (TIF) [file pone.0158315.s002.tif]
