# Supplementary material for: Temporal Changes in BEXSERO® Antigen Sequence Type Associated with Genetic Lineages of Neisseria meningitidis over a 15-Year Period in Western Australia
Source: PLoS One. 2016 Jun 29;11(6):e0158315. doi: 10.1371/journal.pone.0158315 (PMC4927168; doi:10.1371/journal.pone.0158315)
Supplement: S1 Table — (DOCX) [file pone.0158315.s003.docx]

**S1 Table.** Number of cases and number of recovered IMD meningococcal isolates used for sequencing.

| **Year** | **Number of diagnosed cases** | **Number of strains stocked (%)** | **Number of strains sequenced (%)** |
| --- | --- | --- | --- |
| **2000** | 54 | 52 (96.3%) | 48 (92.3%) |
| **2001** | 37 | 34 (91.9%) | 33 (97.1%) |
| **2002** | 58 | 35 (60.3%) | 23 (65.7%) |
| **2003** | 39 | 25 (64.1%) | 15 (60%) |
| **2004** | 38 | 26 (68.4%) | 26 (100%) |
| **2005** | 45 | 24 (53.3%) | 24 (100%) |
| **2006** | 19 | 8 (42.1%) | 8 (100%) |
| **2007** | 20 | 6 (30%) | 6 (100%) |
| **2008** | 23 | 15 (65.2%) | 15 (100%) |
| **2009** | 22 | 18 (81.8%) | 18 (100%) |
| **2010** | 19 | 12 (63.2%) | 12 (100%) |
| **2011** | 22 | 13 (59.1%) | 13 (100%) |
| **2012** | 18 | 12 (66.7%) | 12 (100%) |
| **2013** | 16 | 11 (68.8%) | 11 (100%) |
| **2014** | 17 | 14 (82.4%) | 14 (100%) |
| **Total** | **447** | **305 (68.2%)** | **278 (91.1%)** |
