# Supplementary material for: Temporal Changes in BEXSERO® Antigen Sequence Type Associated with Genetic Lineages of Neisseria meningitidis over a 15-Year Period in Western Australia
Source: PLoS One. 2016 Jun 29;11(6):e0158315. doi: 10.1371/journal.pone.0158315 (PMC4927168; doi:10.1371/journal.pone.0158315)
Supplement: S2 Table — (DOCX) [file pone.0158315.s004.docx]

S**2 Table.** List of meningococcal isolates included in this study including clonal complex and BESERO^®^ antigen typing data.

| **EXNM** | **Age (years)** | **Serogroup** | **Year** | **ST** | **cc** | **fHbp allele** | **fHbp subfamily** | **fHbp peptide** | **NHBA subfamily** | **NadA allele** | **NadA subfamily** | **NadA type** | **PorA type** | **Vaccine coverage^†^** |
| --- | --- | --- | --- | --- | --- | --- | --- | --- | --- | --- | --- | --- | --- | --- |
| 378 | 0.2 | B | 2013 | 897 | no cc | 13 | var1 | fHbp-1.13 | NHBA-8 | - | - | - | P1.12,16 | - |
| 379 | 1.0 | B | 2013 | 10871 | 41/44 | 30 | var3 | fHbp-3.30 | NHBA-2 | - | - | - | P1.7-2,4-13 | + |
| 380 | 64.7 | B | 2013 | 213 | 213 | 505 | var1 | fHbp-1.430 | NHBA-18 | - | - | - | P1.22,14 | + |
| 381 | 3.6 | B | 2012 | 32 | 32 | novel | var3 | fHbp-3.n1 | NHBA-3 | 19 | NadA-1 | NadA-1 | P1.7-2,16-26 | + |
| 382 | 38.2 | B | 2012 | 461 | 461 | 71 | var3 | fHbp-3.47 | NHBA-118 | - | - | - | P1.7-1,4-1 | - |
| 383 | 50.0 | B | 2012 | 7808 | 32 | 29 | var3 | fHbp-3.29 | NHBA-3 | 19 | NadA-1 | NadA-1 | P1.7-2,16-26 | + |
| 384 | 95.0 | B | 2012 | 6058 | 41/44 | 562 | var2 | fHbp-2.486 | NHBA-2 | - | - | - | P1.18-1,34 | + |
| 385 | 0.4 | B | 2012 | 146 | 41/44 | 19 | var2 | fHbp-2.19 | NHBA-43 | - | - | - | P1.22,14-6 | - |
| 386 | 23.5 | B | 2012 | 10571 | 269 | 15 | var1 | fHbp-1.15 | NHBA-21 | - | - | - | P1.7,30-7 | + |
| 387 | 6.4 | B | 2012 | 213 | 213 | 30 | var3 | fHbp-3.30 | NHBA-18 | - | - | - | P1.22,14 | - |
| 388* | 18.7 | B | 2011 | 10493 | 32 | 33 | var3 | fHbp-3.31 | NHBA-3 | 19 | NadA-1 | NadA-1 | P1.7,16-26 | + |
| 389* | 15.3 | B | 2011 | 10871 | 41/44 | 30 | var3 | fHbp-3.30 | NHBA-2 | - | - | - | P1.7-2,4-13 | + |
| 390* | 41.7 | B | 2011 | 437 | 41/44 | 72 | var2 | fHbp-2.19 | NHBA-1 | - | - | - | P1.22-1,14 | + |
| 391* | 64.6 | B | 2011 | 154 | 41/44 | 4 | var1 | fHbp-1.4 | NHBA-2 | - | - | - | P1.7-2,4 | + |
| 392* | 40.0 | B | 2011 | 10541 | 35 | 4 | var1 | fHbp-1.4 | novel | - | - | - | P1.22-1,14 | + |
| 393 | 0.2 | B | 2013 | 944 | 41/44 | 19 | var2 | fHbp-2.19 | NHBA-47 | - | - | - | P1.19,15 | - |
| 394 | 85.0 | W | 2013 | 11 | 11 | 22 | var2 | fHbp-2.22 | NHBA-29 | 5 | NadA-2/3 | NadA-2/3 | P1.5,2 | + |
| 395 | 57.5 | Y | 2012 | 3582 | 23 | 25 | var2 | fHbp-2.25 | NHBA-8 | - | - | - | P1.5-1,2-2 | - |
| 396 | 18.5 | B | 2012 | 213 | 213 | 65 | var3 | fHbp-3.45 | NHBA-18 | - | - | - | P1.22,14 | - |
| 397 | 45.4 | C | 2012 | 11 | 11 | 669 | FS | FS | NHBA-20 | 2 | NadA-2/3 | NadA-2/3 | P1.5-1,10-8 | + |
| 398 | 25.0 | B | 2012 | 60 | 60 | 13 | var1 | fHbp-1.13 | NHBA-18 | - | - | - | P1.5,2 | - |
| 399 | 23.5 | C | 2012 | 212 | 212 | 19 | var2 | fHbp-2.19 | NHBA-47 | - | - | - | P1.19-3,15 | - |
| 400* | 11.8 | B | 2011 | 10493 | 32 | 33 | var3 | fHbp-3.31 | NHBA-3 | 19 | NadA-1 | NadA-1 | P1.7,16-26 | + |
| 401* | 22.6 | B | 2011 | 32 | 32 | 21 | var2 | fHbp-2.21 | NHBA-3 | 19 | NadA-1 | NadA-1 | P1.7,16-26 | + |
| 402 | 75.0 | Y | 2011 | 6799 | 23 | 25 | var2 | fHbp-2.25 | NHBA-8 | - | - | - | P1.5-1,2-2 | - |
| 403 | 53.6 | B | 2011 | 213 | 213 | 505 | var1 | fHbp-1.430 | NHBA-18 | - | - | - | P1.22,14 | + |
| 404* | 51.2 | B | 2011 | 213 | 213 | 505 | var1 | fHbp-1.430 | NHBA-18 | - | - | - | P1.22,14 | + |
| 405* | 83.9 | B | 2011 | 60 | 60 | 13 | var1 | fHbp-1.13 | NHBA-18 | - | - | - | P1.5,2 | - |
| 406* | 0.5 | B | 2011 | 10864 | 269 | 13 | var1 | fHbp-1.13 | NHBA-21 | - | - | - | P1.19,15-1 | - |
| 407 | 70.1 | Y | 2011 | 23 | 23 | 25 | var2 | fHbp-2.25 | NHBA-7 | - | - | - | P1.5-2,10-51 | - |
| 408 | 0.4 | B | 2010 | 3844 | 213 | 33 | var3 | fHbp-3.31 | NHBA-18 | 12 | NadA-4/5 | - | P1.22,14 | - |
| 409 | 46.7 | B | 2010 | novel | 41/44 | 14 | var1 | fHbp-1.14 | NHBA-6 | - | - | - | P1.7-2,4 | + |
| 410* | 20.0 | B | 2010 | 146 | 41/44 | 19 | var2 | fHbp-2.19 | NHBA-43 | - | - | - | P1.22,14-6 | - |
| 411* | 11.2 | B | 2010 | 33 | 32 | 1 | var1 | fHbp-1.1 | NHBA-3 | 1 | NadA-1 | NadA-1 | P1.7-11,30-8 | + |
| 412 | 18.7 | W | 2010 | 1624 | 167 | 24 | var2 | fHbp-2.24 | NHBA-9 | - | - | - | P1.5-1,10-4 | - |
| 413 | 83.8 | C | 2010 | 11 | 11 | 669 | FS | FS | NHBA-20 | 29 | NadA-2/3 | - | P1.5,2 | - |
| 414 | 20.7 | B | 2010 | 146 | 41/44 | 4 | var1 | fHbp-1.4 | NHBA-43 | - | - | - | P1.22,14-6 | + |
| 415* | 1.9 | B | 2010 | 269 | 269 | 15 | var1 | fHbp-1.15 | NHBA-21 | - | - | - | P1.19-1,15-11 | + |
| 416* | 2.0 | B | 2010 | 154 | 41/44 | 4 | var1 | fHbp-1.4 | NHBA-2 | - | - | - | P1.7-2,4 | + |
| 417* | 3.5 | B | 2010 | 32 | 32 | 743 | FS | FS | NHBA-3 | 19 | NadA-1 | NadA-1 | P1.7,16-26 | + |
| 418* | 0.7 | B | 2010 | 146 | 41/44 | 19 | var2 | fHbp-2.19 | NHBA-43 | - | - | - | P1.22,14-6 | - |
| 419 | 36.2 | Y | 2010 | 4183 | 23 | 25 | var2 | fHbp-2.25 | NHBA-8 | - | - | - | P1.5-1,2-2 | - |
| 420* | 40.1 | B | 2009 | 10511 | 41/44 | 19 | var2 | fHbp-2.19 | NHBA-43 | - | - | - | P1.22,14-6 | - |
| 421* | 0.4 | B | 2009 | 1163 | 269 | 19 | var2 | fHbp-2.19 | NHBA-17 | - | - | - | P1.22,9 | - |
| 422* | 0.9 | B | 2009 | 146 | 41/44 | 19 | var2 | fHbp-2.19 | NHBA-43 | - | - | - | P1.22,14-6 | - |
| 423 | 2.4 | B | 2009 | 461 | 461 | 71 | var3 | fHbp-3.47 | NHBA-118 | - | - | - | P1.7-11,30-8 | - |
| 424 | 1.0 | B | 2009 | 1161 | 269 | 13 | var1 | fHbp-1.13 | NHBA-17 | - | - | - | P1.21,9 | - |
| 425 | 83.9 | B | 2009 | 154 | 41/44 | 4 | var1 | fHbp-1.4 | NHBA-2 | - | - | - | P1.5-1,10-4 | + |
| 426 | 10.7 | B | 2009 | 10872 | 41/44 | 13 | var1 | fHbp-1.13 | NHBA-58 | - | - | - | P1.5-2,10-11 | - |
| 427 | 20.0 | B | 2009 | 33 | 32 | 1 | var1 | fHbp-1.1 | NHBA-3 | 1 | NadA-1 | NadA-1 | P1.5-1,10-4 | + |
| 428 | 90.5 | Y | 2009 | 884 | 167 | 23 | var2 | fHbp-2.23 | NHBA-9 | - | - | - | P1.5-1,10-4 | - |
| 429 | 27.5 | C | 2009 | 11 | 11 | 669 | FS | FS | NHBA-20 | 29 | NadA-2/3 | - | P1.5-1,10-8 | - |
| 430 | 17.5 | B | 2009 | 10509 | 41/44 | 4 | var1 | fHbp-1.4 | NHBA-43 | - | - | - | P1.22,14-6 | + |
| 431 | 0.4 | B | 2009 | 10873 | 213 | novel | var1 | fHbp-1.n2 | NHBA-13 | 1 | NadA-1 | NadA-1 | P1.22,14 | + |
| 432 | 0.7 | B | 2009 | 136 | 41/44 | novel | var3 | fHbp-3.n3 | NHBA-10 | - | - | - | P1.17,16-3 | - |
| 433* | 25.3 | B | 2009 | 318 | 41/44 | 14 | var1 | fHbp-1.14 | NHBA-1 | - | - | - | P1.5-2,10-11 | + |
| 434 | 24.2 | C | 2009 | 11 | 11 | 669 | FS | FS | NHBA-20 | 29 | NadA-2/3 | - | P1.5-1,10-8 | - |
| 435* | 12.7 | B | 2009 | 32 | 32 | 33 | var3 | fHbp-3.31 | NHBA-3 | 19 | NadA-1 | NadA-1 | P1.7,16-26 | + |
| 436* | 0.2 | B | 2009 | 575 | 213 | 13 | var1 | fHbp-1.13 | NHBA-18 | - | - | - | P1.22,14 | - |
| 437* | 0.9 | B | 2009 | 3072 | 35 | 16 | var2 | fHbp-2.16 | NHBA-21 | - | - | - | P1.19-1,26 | - |
| 438* | 60.7 | B | 2008 | 32 | 32 | 33 | var3 | fHbp-3.31 | NHBA-3 | 19 | NadA-1 | NadA-1 | P1.7,16-26 | + |
| 439* | 70.4 | B | 2008 | 213 | 213 | 65 | var3 | fHbp-3.45 | novel | 34 | NadA-4/5 | - | P1.22,14 | - |
| 440* | 0.7 | B | 2008 | 146 | 41/44 | 19 | var2 | fHbp-2.19 | NHBA-43 | - | - | - | P1.22,14-6 | - |
| 441* | 17.1 | B | 2008 | 1214 | 269 | novel | var3 | fHbp-3.n4 | novel | - | - | - | P1.19-1,15-11 | - |
| 442* | 32.2 | B | 2008 | 6058 | 41/44 | 19 | var2 | fHbp-2.19 | NHBA-2 | - | - | - | P1.18-1,34 | + |
| 443* | 0.7 | B | 2008 | 213 | 213 | 65 | var3 | fHbp-3.45 | NHBA-18 | 34 | NadA-4/5 | - | P1.18,25 | - |
| 444* | 1.5 | B | 2008 | 318 | 41/44 | 4 | var1 | fHbp-1.4 | NHBA-2 | - | - | - | P1.7-2,4 | + |
| 445* | 1.0 | B | 2008 | 10509 | 41/44 | 4 | var1 | fHbp-1.4 | NHBA-43 | - | - | - | P1.22,14-6 | + |
| 446* | 0.7 | B | 2008 | 146 | 41/44 | 19 | var2 | fHbp-2.19 | NHBA-43 | - | - | - | P1.22,14-6 | - |
| 447* | 0.6 | B | 2008 | 13 | 269 | 19 | var2 | fHbp-2.19 | NHBA-6 | - | - | - | P1.31,16 | - |
| 448* | 21.2 | B | 2008 | 2166 | 269 | 15 | var1 | fHbp-1.15 | NHBA-21 | - | - | - | P1.5-2,10-2 | + |
| 449* | 22.0 | B | 2007 | 318 | 41/44 | 14 | var1 | fHbp-1.14 | NHBA-1 | - | - | - | P1.5-2,10 | + |
| 450* | 18.0 | B | 2007 | 318 | 41/44 | 14 | var1 | fHbp-1.14 | NHBA-1 | - | - | - | P1.5-2,10-11 | + |
| 451* | 93.4 | B | 2007 | 136 | 41/44 | 24 | var2 | fHbp-2.24 | NHBA-10 | - | - | - | P1.17,16-3 | - |
| 452* | 48.6 | B | 2008 | 461 | 461 | 71 | var3 | fHbp-3.47 | NHBA-598 | - | - | - | P1.19-2,13-1 | - |
| 453* | 1.0 | B | 2008 | 10511 | 41/44 | 19 | var2 | fHbp-2.19 | NHBA-43 | - | - | - | P1.22,14-6 | - |
| 454* | 17.6 | B | 2008 | 33 | 32 | 1 | var1 | fHbp-1.1 | NHBA-3 | 1 | NadA-1 | NadA-1 | P1.7-2,4 | + |
| 455* | 20.9 | B | 2008 | 162 | 162 | 1 | var1 | fHbp-1.1 | NHBA-20 | - | - | - | P1.22,14 | + |
| 456 | 7.0 | C | 2006 | 11 | 11 | 134 | var2 | fHbp-2.118 | NHBA-20 | 29 | NadA-2/3 | - | P1.7-2,4 | + |
| 457 | 27.5 | B | 2007 | 1157 | 1157 | 68 | var1 | fHbp-1.13 | NHBA-114 | 20 | NadA-2/3 | - | P1.21-7,16-46 | - |
| 458* | 0.8 | B | 2007 | 146 | 41/44 | 16 | var2 | fHbp-2.16 | NHBA-43 | - | - | - | P1.22,14-6 | - |
| 459* | 60.3 | B | 2007 | 10572 | 32 | 1 | var1 | fHbp-1.1 | NHBA-3 | 1 | NadA-1 | NadA-1 | P1.19,15 | + |
| 460 | 60.6 | B | 2005 | 32 | 32 | 1 | var1 | fHbp-1.1 | NHBA-3 | 1 | NadA-1 | NadA-1 | P1.7,16 | + |
| 461 | 24.8 | B | 2006 | 10545 | 41/44 | 19 | var2 | fHbp-2.19 | NHBA-43 | - | - | - | P1.22,14-6 | - |
| 462 | 0.4 | B | 2006 | 213 | 213 | 33 | var3 | fHbp-3.31 | NHBA-43 | 34 | NadA-4/5 | - | P1.22,14 | - |
| 463 | 65.0 | B | 2006 | 10509 | 41/44 | 4 | var1 | fHbp-1.4 | NHBA-43 | - | - | - | P1.22,14-6 | + |
| 464 | 0.7 | B | 2006 | 146 | 41/44 | 19 | var2 | fHbp-2.19 | NHBA-43 | - | - | - | P1.22,14-6 | - |
| 465 | 0.2 | B | 2006 | 2141 | 32 | 72 | var2 | fHbp-2.19 | NHBA-596 | 5 | NadA-2/3 | NadA-2/3 | P1.5-1,10-1 | + |
| 466 | 14.7 | B | 2006 | 1214 | 269 | 742 | var1 | fHbp-1.622 | novel | - | - | - | P1.19-1,15-11 | + |
| 467 | 2.2 | B | 2006 | novel | no cc | 343 | var1 | fHbp-1.291 | NHBA-21 | - | - | - | P1.7-2,13-1 | + |
| 468 | 1.5 | B | 2005 | 33 | 32 | 1 | var1 | fHbp-1.1 | NHBA-3 | 1 | NadA-1 | NadA-1 | P1.19,15 | + |
| 469 | 1.7 | B | 2005 | 146 | 41/44 | 19 | var2 | fHbp-2.19 | NHBA-43 | - | - | - | P1.22,14-6 | - |
| 470 | 5.2 | B | 2005 | 146 | 41/44 | 19 | var2 | fHbp-2.19 | NHBA-43 | - | - | - | P1.22,14-6 | - |
| 471 | 19.0 | Y | 2005 | 10294 | 23 | 25 | var2 | fHbp-2.25 | NHBA-7 | - | - | - | P1.5-1,10-4 | - |
| 472 | 17.3 | Y | 2005 | 23 | 23 | 25 | var2 | fHbp-2.25 | NHBA-7 | - | - | - | P1.5-1,10-4 | - |
| 473 | 0.4 | B | 2005 | 146 | 41/44 | 19 | var2 | fHbp-2.19 | NHBA-43 | - | - | - | P1.22,14-6 | - |
| 474 | 0.3 | B | 2005 | 275 | 269 | 19 | var2 | fHbp-2.19 | NHBA-17 | - | - | - | P1.22,9 | - |
| 475 | 3.3 | B | 2005 | 146 | 41/44 | 19 | var2 | fHbp-2.19 | NHBA-43 | - | - | - | P1.22,14-6 | - |
| 476 | 20.9 | B | 2005 | 10874 | 41/44 | novel | var2 | fHbp-2.n5 | NHBA-1 | - | - | - | P1.5-2,10-51 | + |
| 477 | 0.6 | B | 2005 | 1214 | 269 | 15 | var1 | fHbp-1.15 | novel | - | - | - | P1.19-1,15-11 | + |
| 478 | 4.0 | B | 2005 | 136 | 41/44 | 24 | var2 | fHbp-2.24 | NHBA-10 | - | - | - | P1.17,16-3 | - |
| 479 | 0.2 | B | 2005 | 318 | 41/44 | 14 | var1 | fHbp-1.14 | NHBA-1 | - | - | - | P1.5-2,10-11 | + |
| 480 | 52.1 | B | 2005 | 146 | 41/44 | 19 | var2 | fHbp-2.19 | NHBA-43 | - | - | - | P1.22,14-6 | - |
| 481 | 2.5 | B | 2005 | 154 | 41/44 | 4 | var1 | fHbp-1.4 | NHBA-2 | - | - | - | P1.7-2,4 | + |
| 482 | 0.3 | B | 2005 | 7516 | 41/44 | novel | var1 | fHbp-1.n6 | NHBA-2 | - | - | - | P1.12-6,13-4 | + |
| 483 | 0.7 | B | 2005 | 47 | 41/44 | 19 | var2 | fHbp-2.19 | NHBA-32 | - | - | - | P1.7-2,13-1 | - |
| 484 | 48.6 | B | 2005 | 42 | 41/44 | 110 | var1 | fHbp-1.110 | NHBA-2 | - | - | - | P1.18-4,25 | + |
| 485 | 17.5 | B | 2005 | 1161 | 269 | 13 | var1 | fHbp-1.13 | NHBA-191 | - | - | - | P1.22,9 | - |
| 486 | 3.3 | B | 2005 | 10875 | 32 | 1 | var1 | fHbp-1.1 | NHBA-3 | 1 | NadA-1 | NadA-1 | P1.19,15 | + |
| 487 | 1.1 | B | 2005 | 7516 | 41/44 | novel | var1 | fHbp-1.n7 | NHBA-2 | - | - | - | P1.12-6,13-4 | + |
| 488 | 4.7 | B | 2005 | 639 | 32 | 1 | var1 | fHbp-1.1 | NHBA-3 | 1 | NadA-1 | NadA-1 | P1.7,30-7 | + |
| 489 | 42.8 | B | 2005 | 213 | 213 | 13 | var1 | fHbp-1.13 | NHBA-18 | 34 | NadA-4/5 | - | P1.22,14 | - |
| 490 | 0.1 | B | 2005 | 146 | 41/44 | 19 | var2 | fHbp-2.19 | NHBA-43 | - | - | - | P1.22,14-6 | - |
| 491 | 62.7 | B | 2004 | 639 | 32 | 1 | var1 | fHbp-1.1 | NHBA-3 | 1 | NadA-1 | NadA-1 | P1.7,30-3 | + |
| 492 | 20.0 | C | 2004 | 11 | 11 | 1 | var1 | fHbp-1.1 | NHBA-20 | 29 | NadA-2/3 | - | P1.5-1,10-1 | + |
| 493 | 20.9 | C | 2004 | 5169 | 11 | 92 | var2 | fHbp-2.95 | NHBA-20 | 29 | NadA-2/3 | - | P1.5-1,10-4 | - |
| 494 | 0.8 | B | 2004 | 146 | 41/44 | 19 | var2 | fHbp-2.19 | NHBA-43 | - | - | - | P1.22,14-6 | - |
| 495 | 0.1 | B | 2004 | 33 | 32 | 1 | var1 | fHbp-1.1 | NHBA-3 | 1 | NadA-1 | NadA-1 | P1.22-1,14 | + |
| 496 | 18.9 | B | 2004 | 318 | 41/44 | novel | var1 | fHbp-1.n8 | NHBA-1 | - | - | - | P1.5-2,10-11 | + |
| 497 | 27.3 | C | 2004 | 11 | 11 | 134 | var2 | fHbp-2.118 | NHBA-20 | 19 | NadA-1 | NadA-1 | P1.7-2,4 | + |
| 498 | 1.1 | B | 2004 | 1788 | 41/44 | 19 | var2 | fHbp-2.19 | NHBA-43 | - | - | - | P1.22,14-6 | - |
| 499 | 17.9 | B | 2004 | 10876 | 41/44 | novel | var1 | fHbp-1.n9 | NHBA-1 | - | - | - | P1.5-2,10-11 | + |
| 500 | 29.2 | B | 2004 | 639 | 32 | 1 | var1 | fHbp-1.1 | NHBA-3 | 1 | NadA-1 | NadA-1 | P1.7,30-7 | + |
| 501 | 3.0 | B | 2004 | 146 | 41/44 | 19 | var2 | fHbp-2.19 | NHBA-43 | - | - | - | P1.22,14-6 | - |
| 502 | 17.5 | B | 2004 | 1214 | 269 | 742 | var1 | fHbp-1.622 | novel | - | - | - | P1.19-1,15-11 | + |
| 503 | 77.3 | W | 2004 | 2725 | 22 | 19 | var2 | fHbp-2.19 | NHBA-20 | - | - | - | P1.18-1,3 | - |
| 504 | 81.4 | B | 2004 | 9611 | 32 | 66 | var1 | fHbp-1.4 | NHBA-255 | 19 | NadA-1 | NadA-1 | P1.7,16-26 | + |
| 505 | 8.9 | C | 2004 | novel | 11 | 11 | var1 | fHbp-1.11 | NHBA-20 | 2 | NadA-2/3 | NadA-2/3 | P1.5-1,10-1 | + |
| 506 | 0.3 | B | 2004 | 146 | 41/44 | 19 | var2 | fHbp-2.19 | NHBA-43 | - | - | - | P1.22,14-6 | - |
| 507 | 3.9 | B | 2004 | 13 | 269 | 19 | var2 | fHbp-2.19 | NHBA-6 | - | - | - | P1.31,16 | - |
| 508 | 1.7 | B | 2004 | 46 | 41/44 | 4 | var1 | fHbp-1.4 | NHBA-2 | - | - | - | P1.7-2,4 | + |
| 509 | 32.5 | B | 2004 | 146 | 41/44 | 19 | var2 | fHbp-2.19 | NHBA-43 | - | - | - | P1.22,14-6 | - |
| 510 | 4.8 | B | 2004 | 33 | 32 | 1 | var1 | fHbp-1.1 | NHBA-3 | 1 | NadA-1 | NadA-1 | P1.19,15 | + |
| 511 | 18.7 | B | 2004 | 154 | 41/44 | 4 | var1 | fHbp-1.4 | NHBA-2 | - | - | - | P1.7-2,4 | + |
| 512 | 26.0 | B | 2004 | 41 | 41/44 | 4 | var1 | fHbp-1.4 | NHBA-2 | - | - | - | P1.7-2,4 | + |
| 513 | 22.2 | C | 2004 | 11 | 11 | 134 | var2 | fHbp-2.118 | NHBA-20 | 2 | NadA-2/3 | NadA-2/3 | P1.7-2,4 | + |
| 514 | 14.9 | B | 2004 | 318 | 41/44 | 97 | var1 | fHbp-1.89 | NHBA-1 | - | - | - | P1.5-2,10-11 | + |
| 515 | 16.5 | B | 2004 | novel | no cc | 711 | var1 | fHbp-1.605 | NHBA-24 | - | - | - | P1.21,16 | + |
| 516 | 19.0 | B | 2004 | 136 | 41/44 | 24 | var2 | fHbp-2.24 | NHBA-10 | - | - | - | P1.17,16-3 | - |
| 517 | 73.1 | B | 2003 | 42 | 41/44 | 112 | var3 | fHbp-3.111 | NHBA-2 | - | - | - | P1.7-2,4 | + |
| 518 | 3.9 | B | 2003 | 1791 | 269 | 15 | var1 | fHbp-1.15 | NHBA-21 | - | - | - | P1.19-1,15-11 | + |
| 519 | 2.6 | B | 2003 | 146 | 41/44 | 19 | var2 | fHbp-2.19 | NHBA-43 | - | - | - | P1.22,14-6 | - |
| 520 | 1.2 | B | 2003 | 146 | 41/44 | 19 | var2 | fHbp-2.19 | NHBA-43 | - | - | - | P1.22,14-6 | - |
| 521 | 3.4 | B | 2003 | 146 | 41/44 | 16 | var2 | fHbp-2.16 | NHBA-43 | - | - | - | P1.22,14-6 | - |
| 522 | 6.0 | B | 2003 | 1791 | 269 | 15 | var1 | fHbp-1.15 | NHBA-21 | - | - | - | P1.19-1,15-11 | + |
| 523 | 36.5 | B | 2003 | 170 | 41/44 | 19 | var2 | fHbp-2.19 | NHBA-116 | - | - | - | P1.5-1,10-4 | - |
| 524 | 17.7 | B | 2003 | 146 | 41/44 | 1 | var1 | fHbp-1.1 | NHBA-43 | - | - | - | P1.22,14-6 | + |
| 525 | 21.3 | B | 2003 | 1157 | 1157 | 546 | var1 | fHbp-1.456 | NHBA-114 | 20 | NadA-2/3 | - | P1.21-7,16 | + |
| 526 | 16.1 | B | 2002 | 33 | 32 | 74 | var2 | fHbp-2.16 | NHBA-3 | 1 | NadA-1 | NadA-1 | P1.19,15 | + |
| 527 | 0.9 | B | 2002 | 6566 | 41/44 | 19 | var2 | fHbp-2.19 | NHBA-43 | - | - | - | P1.22,14-6 | - |
| 528 | 19.4 | C | 2002 | 11 | 11 | 11 | var1 | fHbp-1.11 | NHBA-20 | 29 | NadA-2/3 | - | P1.5-1,10-1 | + |
| 529 | 0.9 | B | 2002 | 146 | 41/44 | 713 | var1 | fHbp-1.8 | NHBA-43 | - | - | - | P1.22,14-6 | + |
| 530 | 42.6 | B | 2002 | 46 | 41/44 | 4 | var1 | fHbp-1.4 | NHBA-2 | - | - | - | P1.7-2,4 | + |
| 531 | 16.3 | B | 2002 | 42 | 41/44 | 112 | var3 | fHbp-3.111 | NHBA-2 | - | - | - | P1.7-2,4 | + |
| 532 | 18.8 | B | 2002 | 41 | 41/44 | 4 | var1 | fHbp-1.4 | NHBA-2 | - | - | - | P1.19,15 | + |
| 533 | 1.2 | B | 2002 | 41 | 41/44 | 4 | var1 | fHbp-1.4 | NHBA-2 | - | - | - | P1.7-2,4 | + |
| 534 | 18.1 | B | 2002 | 42 | 41/44 | 112 | var3 | fHbp-3.111 | NHBA-2 | - | - | - | P1.7-2,4 | + |
| 535 | 18.8 | B | 2002 | 318 | 41/44 | 281 | var1 | fHbp-1.14 | NHBA-1 | - | - | - | P1.5-2,10-11 | + |
| 536 | 24.2 | C | 2002 | 11 | 11 | 134 | var2 | fHbp-2.118 | NHBA-20 | 5 | NadA-2/3 | NadA-2/3 | P1.7-2,4-13 | + |
| 537 | 0.7 | B | 2000 | 154 | 41/44 | 4 | var1 | fHbp-1.4 | NHBA-2 | - | - | - | P1.7-2,4-13 | + |
| 538 | 25.0 | C | 2000 | 11 | 11 | - | None | fhbp-1,2,3-hybrid | NHBA-20 | 2 | NadA-2/3 | NadA-2/3 | P1.5,2 | + |
| 540 | 18.6 | B | 2000 | 5709 | 35 | 4 | var1 | fHbp-1.4 | NHBA-21 | - | - | - | P1.19-1,26 | + |
| 541 | 1.5 | B | 2000 | 4711 | 41/44 | 19 | var2 | fHbp-2.19 | NHBA-43 | - | - | - | P1.22,14-6 | - |
| 542 | 18.8 | B | 2000 | 32 | 32 | 1 | var1 | fHbp-1.1 | NHBA-3 | 1 | NadA-1 | NadA-1 | P1.7,16 | + |
| 543 | 1.6 | C | 2000 | 11 | 11 | - | None | fhbp-1,2,3-hybrid | NHBA-20 | 3 | NadA-2/3 | NadA-2/3 | P1.5,2 | + |
| 544 | 1.0 | B | 2000 | 34 | 32 | 1 | var1 | fHbp-1.1 | NHBA-489 | 1 | NadA-1 | NadA-1 | P1.22,15 | + |
| 545 | 21.6 | B | 2000 | 32 | 32 | 1 | var1 | fHbp-1.1 | NHBA-3 | 1 | NadA-1 | NadA-1 | P1.7,16 | + |
| 546 | 32.9 | C | 2000 | 11 | 11 | 36 | var1 | fHbp-1.37 | NHBA-20 | 29 | NadA-2/3 | - | P1.5,2 | + |
| 547 | 85.5 | B | 2000 | 32 | 32 | 13 | var1 | fHbp-1.13 | NHBA-3 | 19 | NadA-1 | NadA-1 | P1.7-2,16-12 | + |
| 548 | 45.1 | C | 2000 | 11 | 11 | 1 | var1 | fHbp-1.1 | NHBA-20 | 29 | NadA-2/3 | - | P1.18-1,3 | + |
| 549 | 4.1 | B | 2000 | 146 | 41/44 | 19 | var2 | fHbp-2.19 | NHBA-43 | - | - | - | P1.22,14-6 | - |
| 550 | 1.8 | C | 2000 | 11 | 11 | 11 | var1 | fHbp-1.11 | NHBA-20 | 2 | NadA-2/3 | NadA-2/3 | P1.5-1,10-8 | + |
| 551 | 3.7 | B | 2000 | novel | no cc | 757 | var1 | fHbp-1.628 | NHBA-255 | - | - | - | P1.7-1,1 | + |
| 552 | 1.7 | B | 2000 | 154 | 41/44 | 4 | var1 | fHbp-1.4 | NHBA-2 | - | - | - | P1.7-2,4 | + |
| 553 | 17.5 | C | 2000 | 11 | 11 | 715 | var1 | fHbp-1.606 | NHBA-19 | 29 | NadA-2/3 | - | P1.18-1,3 | + |
| 554 | 0.2 | B | 2000 | 146 | 41/44 | 19 | var2 | fHbp-2.19 | NHBA-43 | - | - | - | P1.22,14-6 | - |
| 555 | 85.9 | B | 2000 | novel | 41/44 | 4 | var1 | fHbp-1.4 | NHBA-2 | - | - | - | P1.7-2,4 | + |
| 556 | 0.4 | B | 2000 | 146 | 41/44 | 19 | var2 | fHbp-2.19 | NHBA-43 | - | - | - | P1.22,14-6 | - |
| 557 | 14.6 | B | 2000 | 33 | 32 | 1 | var1 | fHbp-1.1 | NHBA-3 | - | - | - | P1.19,15 | + |
| 558 | 57.9 | B | 2000 | 213 | 213 | 65 | var3 | fHbp-3.45 | NHBA-18 | 34 | NadA-4/5 | - | P1.22,14 | - |
| 559 | 21.0 | C | 2000 | 11 | 11 | 36 | var1 | fHbp-1.37 | NHBA-20 | 29 | NadA-2/3 | - | P1.5,2 | + |
| 560 | 1.3 | B | 2000 | 146 | 41/44 | 19 | var2 | fHbp-2.19 | NHBA-43 | - | - | - | P1.22,14-6 | - |
| 561 | 6.2 | B | 2000 | novel | no cc | 136 | var2 | fHbp-2.119 | NHBA-255 | - | - | - | P1.7-1,1 | - |
| 562 | 0.2 | B | 2000 | 146 | 41/44 | 19 | var2 | fHbp-2.19 | NHBA-43 | - | - | - | P1.22,14-6 | - |
| 563 | 19.6 | B | 2000 | 41 | 41/44 | 16 | var2 | fHbp-2.16 | NHBA-2 | - | - | - | P1.7-2,4 | + |
| 564 | 64.7 | B | 2000 | 46 | 41/44 | 4 | var1 | fHbp-1.4 | NHBA-2 | - | - | - | P1.7-2,4 | + |
| 565 | 18.9 | B | 2000 | 32 | 32 | 1 | var1 | fHbp-1.1 | NHBA-3 | 1 | NadA-1 | NadA-1 | P1.7,16 | + |
| 566 | 1.7 | B | 2000 | 32 | 32 | 21 | var2 | fHbp-2.21 | NHBA-3 | 19 | NadA-1 | NadA-1 | P1.7,16-26 | + |
| 567 | 1.5 | B | 2000 | 283 | 269 | 15 | var1 | fHbp-1.15 | NHBA-21 | - | - | - | P1.19-1,15-11 | + |
| 568 | 7.1 | B | 2000 | 146 | 41/44 | 19 | var2 | fHbp-2.19 | NHBA-43 | - | - | - | P1.22,14-6 | - |
| 569 | 5.8 | B | 2000 | novel | 41/44 | 113 | var3 | fHbp-3.111 | NHBA-116 | - | - | - | P1.7-2,13-1 | - |
| 570 | 1.3 | C | 2000 | 11 | 11 | 11 | var1 | fHbp-1.11 | NHBA-20 | 29 | NadA-2/3 | - | P1.5-1,10-8 | + |
| 571 | 12.7 | B | 2000 | 318 | 41/44 | 14 | var1 | fHbp-1.14 | NHBA-1 | - | - | - | P1.5-2,10-11 | + |
| 572 | 0.5 | B | 2000 | 146 | 41/44 | 19 | var2 | fHbp-2.19 | NHBA-43 | - | - | - | P1.22,14-6 | - |
| 573 | 2.9 | C | 2000 | 8 | 8 | 16 | var2 | fHbp-2.16 | NHBA-21 | 6 | NadA-2/3 | NadA-2/3 | P1.5,2 | + |
| 574 | 9.9 | B | 2001 | 146 | 41/44 | 19 | var2 | fHbp-2.19 | NHBA-43 | - | - | - | P1.22,14-6 | - |
| 575 | 5.8 | B | 2001 | 5682 | 32 | 1 | var1 | fHbp-1.1 | NHBA-3 | 1 | NadA-1 | NadA-1 | P1.19-1,26 | + |
| 576 | 6.3 | B | 2001 | 33 | 32 | 1 | var1 | fHbp-1.1 | NHBA-3 | 1 | NadA-1 | NadA-1 | P1.19,15 | + |
| 577 | 17.5 | B | 2001 | 42 | 41/44 | 4 | var1 | fHbp-1.4 | NHBA-571 | - | - | - | P1.19-1,15-35 | + |
| 578 | 0.4 | B | 2001 | 146 | 41/44 | 19 | var2 | fHbp-2.19 | NHBA-43 | - | - | - | P1.19-3,15 | - |
| 579 | 6.7 | C | 2001 | 11 | 11 | 505 | var1 | fHbp-1.430 | novel | 3 | NadA-2/3 | NadA-2/3 | ND | + |
| 580 | 0.4 | B | 2001 | 146 | 41/44 | 19 | var2 | fHbp-2.19 | NHBA-43 | - | - | - | P1.22,14-6 | - |
| 581 | 11.0 | B | 2001 | 146 | 41/44 | 19 | var2 | fHbp-2.19 | NHBA-43 | - | - | - | P1.22,14-6 | - |
| 582 | 2.1 | B | 2001 | 41 | 41/44 | 1 | var1 | fHbp-1.1 | NHBA-2 | - | - | - | P1.7-2,4 | + |
| 583 | 0.3 | B | 2001 | 146 | 41/44 | 19 | var2 | fHbp-2.19 | NHBA-43 | - | - | - | P1.22,14-6 | - |
| 584 | 0.2 | B | 2001 | 146 | 41/44 | 19 | var2 | fHbp-2.19 | NHBA-43 | - | - | - | P1.22,14-6 | - |
| 585 | 5.6 | B | 2001 | 2012 | 41/44 | 787 | var1 | fHbp-1.650 | NHBA-116 | - | - | - | P1.5-1,10-10 | + |
| 586 | 2.7 | C | 2001 | 212 | 212 | 828 | var1 | fHbp-1.697 | NHBA-47 | - | - | - | P1.19-3,15 | + |
| 587 | 0.5 | B | 2001 | 7394 | 41/44 | 4 | var1 | fHbp-1.4 | NHBA-2 | - | - | - | P1.7-2,4 | + |
| 588 | 11.4 | B | 2001 | 34 | 32 | 1 | var1 | fHbp-1.1 | NHBA-3 | 1 | NadA-1 | NadA-1 | P1.22,14-6 | + |
| 589 | 3.1 | B | 2001 | 41 | 41/44 | 1 | var1 | fHbp-1.1 | NHBA-2 | - | - | - | P1.7-2,4 | + |
| 590 | 1.4 | B | 2001 | novel | no cc | 71 | var3 | fHbp-3.47 | NHBA-596 | - | - | - | P1.18-1,3 | - |
| 591 | 1.4 | B | 2001 | novel | 41/44 | 101 | var2 | fHbp-2.101 | NHBA-1 | - | - | - | P1.5-2,10-51 | + |
| 592 | 3.3 | B | 2001 | 42 | 41/44 | 112 | var3 | fHbp-3.111 | NHBA-2 | - | - | - | P1.7-2,4 | + |
| 593 | 18.9 | C | 2001 | 11 | 11 | 11 | var1 | fHbp-1.11 | NHBA-20 | 2 | NadA-2/3 | NadA-2/3 | P1.5-1,10-8 | + |
| 594 | 42.3 | C | 2001 | 11 | 11 | 134 | var2 | fHbp-2.118 | NHBA-20 | 29 | NadA-2/3 | - | P1.7-2,4 | + |
| 595 | 0.7 | B | 2001 | 32 | 32 | 1 | var1 | fHbp-1.1 | NHBA-3 | 1 | NadA-1 | NadA-1 | P1.7,16 | + |
| 596 | 0.5 | C | 2001 | 11 | 11 | 11 | var1 | fHbp-1.11 | NHBA-20 | 2 | NadA-2/3 | NadA-2/3 | P1.5-1,10-8 | + |
| 597 | 14.4 | B | 2001 | 42 | 41/44 | 112 | var3 | fHbp-3.111 | NHBA-2 | - | - | - | P1.7-2,4 | + |
| 598 | 18.4 | B | 2001 | 1163 | 269 | 19 | var2 | fHbp-2.19 | NHBA-17 | - | - | - | P1.22,9 | - |
| 599 | 0.9 | B | 2001 | 283 | 269 | 15 | var1 | fHbp-1.15 | NHBA-21 | - | - | - | P1.7-2,16 | + |
| 600 | 2.3 | B | 2001 | 213 | 213 | 746 | FS | FS | NHBA-18 | 34 | NadA-4/5 | - | P1.22,14 | - |
| 601 | 18.3 | B | 2001 | 46 | 41/44 | 4 | var1 | fHbp-1.4 | NHBA-2 | - | - | - | P1.7-2,4 | + |
| 602 | 1.0 | B | 2001 | 32 | 32 | 1 | var1 | fHbp-1.1 | NHBA-3 | 1 | NadA-1 | NadA-1 | P1.7,16 | + |
| 603 | 58.5 | B | 2001 | 343 | 32 | 1 | var1 | fHbp-1.1 | NHBA-3 | 1 | NadA-1 | NadA-1 | P1.7,16-26 | + |
| 604 | 2.5 | B | 2001 | 32 | 32 | 29 | var3 | fHbp-3.29 | NHBA-3 | 19 | NadA-1 | NadA-1 | P1.7,16-26 | + |
| 605 | 20.9 | B | 2014 | 9193 | 213 | 505 | var1 | fHbp-1.430 | NHBA-18 | - | - | - | P1.22,14 | + |
| 606 | 0.4 | C | 2014 | 11 | 11 | 669 | FS | FS | NHBA-20 | 29 | NadA-2/3 | - | P1.5-1,10-8 | - |
| 607 | 21.4 | C | 2014 | 11 | 11 | 710 | var1 | fHbp-1.601 | NHBA-20 | 29 | NadA-2/3 | - | P1.5-1,10-8 | + |
| 608 | 7.8 | B | 2014 | 461 | 461 | 71 | var3 | fHbp-3.47 | NHBA-118 | - | - | - | P1.19-2,13-1 | - |
| 609 | 0.0 | B | 2014 | 191 | 41/44 | 19 | var2 | fHbp-2.19 | NHBA-43 | - | - | - | P1.22,13-9 | - |
| 610 | 74.3 | B | 2014 | 1825 | 213 | 65 | var3 | fHbp-3.45 | NHBA-18 | - | - | - | P1.22,14 | - |
| 611 | 0.6 | B | 2014 | novel | 35 | 101 | var2 | fHbp-2.101 | NHBA-5 | - | - | - | P1.22,14 | - |
| 612 | 19.0 | W | 2014 | 11 | 11 | 22 | var2 | fHbp-2.22 | NHBA-29 | 5 | NadA-2/3 | NadA-2/3 | P1.5,2 | + |
| 613 | 19.2 | B | 2014 | 41 | 41/44 | 778 | var3 | fHbp-3.499 | NHBA-2 | - | - | - | P1.7,30-5 | + |
| 614 | 84.0 | Y | 2014 | 23 | 23 | 25 | var2 | fHbp-2.25 | NHBA-7 | - | - | - | P1.5-1,10-4 | - |
| 615 | 22.7 | B | 2014 | 213 | 213 | 505 | var1 | fHbp-1.430 | NHBA-18 | - | - | - | P1.22,14 | + |
| 616 | 23.4 | C | 2013 | 11 | 11 | 11 | var1 | fHbp-1.11 | NHBA-20 | 29 | NadA-2/3 | - | P1.5-1,10-8 | + |
| 617 | 32.6 | B | 2013 | 213 | 213 | 505 | var1 | fHbp-1.430 | NHBA-18 | - | - | - | P1.22,14 | + |
| 618 | 19.2 | B | 2013 | 213 | 213 | 65 | var3 | fHbp-3.45 | NHBA-18 | - | - | - | P1.22,14 | - |
| 619 | 57.2 | C | 2013 | 11 | 11 | 10 | var1 | fHbp-1.10 | NHBA-20 | 29 | NadA-2/3 | - | P1.5-1,10-8 | + |
| 620 | 26.2 | B | 2013 | 213 | 213 | 505 | var1 | fHbp-1.430 | NHBA-18 | - | - | - | P1.22,14 | + |
| 621 | 46.8 | Y | 2013 | 23 | 23 | 25 | var2 | fHbp-2.25 | NHBA-7 | - | - | - | P1.5-1,10-4 | - |
| 622 | 0.5 | B | 2003 | 32 | 32 | 56 | var1 | fHbp-1.69 | NHBA-3 | 19 | NadA-1 | NadA-1 | P1.7,16-26 | + |
| 623 | 32.4 | B | 2003 | 146 | 41/44 | 134 | var2 | fHbp-2.118 | NHBA-43 | - | - | - | P1.22,14-6 | - |
| 624 | 19.4 | C | 2003 | 11 | 11 | 134 | var2 | fHbp-2.118 | NHBA-20 | 29 | NadA-2/3 | - | P1.7-2,4 | + |
| 625 | 3.5 | B | 2003 | novel | 41/44 | 4 | var1 | fHbp-1.4 | NHBA-2 | - | - | - | P1.7-2,4 | + |
| 626 | 1.4 | B | 2003 | 2266 | 41/44 | 1 | var1 | fHbp-1.1 | NHBA-214 | - | - | - | P1.7-2,4 | + |
| 627 | 2.8 | B | 2003 | 798 | 269 | 713 | var1 | fHbp-1.8 | NHBA-32 | - | - | - | P1.7-1,1 | + |
| 628 | 4.2 | C | 2002 | 212 | 212 | 33 | var3 | fHbp-3.31 | NHBA-47 | - | - | - | P1.19-3,15 | - |
| 629 | 4.1 | B | 2002 | 146 | 41/44 | 19 | var2 | fHbp-2.19 | NHBA-43 | - | - | - | P1.22,14-6 | - |
| 630 | 0.2 | B | 2002 | 146 | 41/44 | 19 | var2 | fHbp-2.19 | NHBA-43 | - | - | - | P1.22,14-6 | - |
| 631 | 11.3 | C | 2002 | 11 | 11 | 11 | var1 | fHbp-1.11 | NHBA-20 | 2 | NadA-2/3 | NadA-2/3 | P1.5-1,10-8 | + |
| 632 | 1.5 | B | 2002 | 41 | 41/44 | 16 | var2 | fHbp-2.16 | NHBA-2 | - | - | - | P1.7-2,4 | + |
| 633 | 0.2 | B | 2002 | 146 | 41/44 | 713 | var1 | fHbp-1.8 | NHBA-43 | - | - | - | P1.22,14-6 | + |
| 634 | 10.3 | B | 2002 | 32 | 32 | 1 | var1 | fHbp-1.1 | NHBA-3 | 1 | NadA-1 | NadA-1 | P1.7,16 | + |
| 635 | 0.9 | B | 2002 | 146 | 41/44 | 19 | var2 | fHbp-2.19 | NHBA-43 | - | - | - | P1.22,14-6 | - |
| 636 | 42.5 | B | 2002 | 32 | 32 | 1 | var1 | fHbp-1.1 | NHBA-3 | 1 | NadA-1 | NadA-1 | P1.7,16 | + |
| 637 | 28.6 | B | 2002 | novel | 41/44 | 733 | var1 | fHbp-1.246 | NHBA-26 | - | - | - | P1.5-1,2-2 | + |
| 638 | 23.6 | B | 2002 | 32 | 32 | 33 | var3 | fHbp-3.31 | NHBA-3 | 19 | NadA-1 | NadA-1 | P1.7,16-26 | + |
| 639 | 0.5 | B | 2002 | 1194 | 41/44 | 4 | var1 | fHbp-1.4 | NHBA-2 | - | - | - | P1.18-1,3 | + |
| 640 | 0.3 | W | 2001 | 1061 | 22 | 16 | var2 | fHbp-2.16 | NHBA-20 | - | - | - | P1.18-1,3 | - |
| 641 | 0.4 | B | 2000 | 2172 | no cc | 25 | var2 | fHbp-2.25 | NHBA-129 | - | - | - | ND | - |
| 642 | 0.7 | B | 2014 | novel | 213 | 33 | var3 | fHbp-3.31 | NHBA-7 | 12 | NadA-4/5 | - | P1.22,14 | - |
| 643 | 0.5 | B | 2014 | 154 | 41/44 | 4 | var1 | fHbp-1.4 | NHBA-2 | - | - | - | P1.7-2,4 | + |
| 644 | 20.0 | B | 2000 | 11 | 11 | 36 | var1 | fHbp-1.37 | NHBA-20 | 29 | NadA-2/3 | - | P1.5,2 | + |
| 645 | 73.6 | B | 2000 | novel | 41/44 | 4 | var1 | fHbp-1.4 | NHBA-2 | - | - | - | P1.7-2,4-13 | + |
| 646 | 2.4 | B | 2000 | 6871 | 32 | 1 | var1 | fHbp-1.1 | NHBA-3 | 1 | NadA-1 | NadA-1 | P1.7,16-26 | + |
| 647 | 0.6 | B | 2000 | 32 | 32 | 1 | var1 | fHbp-1.1 | NHBA-3 | 1 | NadA-1 | NadA-1 | P1.7,16 | + |
| 648 | 5.4 | B | 2000 | 32 | 32 | 1 | var1 | fHbp-1.1 | NHBA-3 | 1 | NadA-1 | NadA-1 | P1.7,16 | + |
| 649 | 62.0 | B | 2000 | 146 | 41/44 | 19 | var2 | fHbp-2.19 | NHBA-43 | - | - | - | P1.22,14-6 | - |
| 650 | 20.1 | C | 2000 | 11 | 11 | 669 | FS | FS | NHBA-20 | 29 | NadA-2/3 | - | P1.18-1,3 | - |
| 651 | 10.1 | B | 2000 | 33 | 32 | 1 | var1 | fHbp-1.1 | NHBA-3 | 1 | NadA-1 | NadA-1 | P1.19,15 | + |
| 652 | 17.9 | W | 2014 | 11 | 11 | 22 | var2 | fHbp-2.22 | NHBA-29 | 5 | NadA-2/3 | NadA-2/3 | P1.5,2 | + |
| 653 | 33.5 | B | 2001 | 41 | 41/44 | 4 | var1 | fHbp-1.4 | NHBA-2 | - | - | - | P1.7-2,4 | + |
| 654 | 18.9 | B | 2000 | 146 | 41/44 | 1 | var1 | fHbp-1.1 | NHBA-43 | - | - | - | P1.7-2,4 | + |
| 655 | 0.0 | W | 2000 | 44 | 41/44 | 713 | var1 | fHbp-1.8 | NHBA-2 | - | - | - | P1.19-1,26 | + |
| 656 | 21.2 | B | 2000 | 921 | 41/44 | 19 | var2 | fHbp-2.19 | NHBA-2 | - | - | - | P1.19,15-1 | + |

FS: Frameshift; ND: Not determined

* indicates isolates used in the MATS ELISA assay

**^†^** Isolates were predicted to be covered by the BEXSERO^®^ vaccine if they possessed intact genes for one or more of the following antigenic variants: all fHbp-1 except fHbp-1.13, NHBA-1, NHBA-2, NadA-1, NadA-2/3 or P1.4 PorA subfamily.
