# Supplementary material for: Temporal Changes in BEXSERO® Antigen Sequence Type Associated with Genetic Lineages of Neisseria meningitidis over a 15-Year Period in Western Australia
Source: PLoS One. 2016 Jun 29;11(6):e0158315. doi: 10.1371/journal.pone.0158315 (PMC4927168; doi:10.1371/journal.pone.0158315)
Supplement: S3 Table — (DOCX) [file pone.0158315.s005.docx]

**S3 Table.** Genetic diversity of *N. meningitidis* isolates in this study.

| **Serogroup** | **Clonal complex** | **No. of isolates** | **No. of STs** | **ST present more than twice (frequency)** |
| --- | --- | --- | --- | --- |
| B (n=227) | cc41/44 | 120 | 30 | ST-146 (44)  ST-41 (9)  ST-318 (9)  ST-154 (8)  ST-42 (7)  ST-46 (4)  ST-136 (4)  ST-10509 (3) |
|  | cc32 | 45 | 13 | ST-32 (20)  ST-33 (10)  ST-639 (3) |
|  | cc213 | 21 | 7 | ST-213 (15) |
|  | cc269 | 20 | 12 | ST-1214 (4) |
|  | cc35 | 4 | 4 | - |
|  | cc461 | 4 | 1 | ST-461 (4) |
|  | cc60 | 2 | 1 | - |
|  | cc1157 | 2 | 1 | - |
|  | cc11 | 1 | 1 | - |
|  | cc162 | 1 | 1 | - |
|  | unassigned | 7 | 7 | - |
|  |  |  |  |  |
| C (n=35) | cc11 | 31 | 3 | ST-11 (29) |
|  | cc212 | 3 | 1 | ST-212 (3) |
|  | cc8 | 1 | 1 | - |
|  |  |  |  |  |
| W (n=7) | cc11 | 3 | 1 | ST-11 (3) |
|  | cc22 | 2 | 2 | - |
|  | cc167 | 1 | 1 | - |
|  | cc41/44 | 1 | 1 | - |
|  |  |  |  |  |
| Y (n=9) | cc23 | 8 | 5 | ST-23(4) |
|  | cc167 | 1 | 1 | - |
