# Supplementary material for: Temporal Changes in BEXSERO® Antigen Sequence Type Associated with Genetic Lineages of Neisseria meningitidis over a 15-Year Period in Western Australia
Source: PLoS One. 2016 Jun 29;11(6):e0158315. doi: 10.1371/journal.pone.0158315 (PMC4927168; doi:10.1371/journal.pone.0158315)
Supplement: S4 Table — (DOCX) [file pone.0158315.s006.docx]

**S4 Table.** Data for the MATS ELISA assay performed in this study.

|  |  |  |  | **MATS RP** | | |  | **MATS coverage**** | | | | | **Covered antigens** | |  |
| --- | --- | --- | --- | --- | --- | --- | --- | --- | --- | --- | --- | --- | --- | --- | --- |
| **EXNM** | **year** | **ST** | **cc** | **NHBA** | **fHbp** | **NadA** | **PorA P1.4** | **Overall** | **NHBA** | **fHbp** | **NadA** | **PorA** | **No. of antigens** | **Antigen combination** | **Cluster** |
| 388 | 2011 | 10493 | 32 | 0.329 | 0 | 0.0002 | no | yes | yes | no | no | no | 1 | NHBA | 3 |
| 389 | 2011 | 10871 | 41/44 | 1.012 | 0 | 0 | yes | yes | yes | no | no | yes | 2 | NHBA+PorA | 1b |
| 390 | 2011 | 437 | 41/44 | 0.715 | 0.0005 | 0 | no | yes | yes | no | no | no | 1 | NHBA | - |
| 391 | 2011 | 154 | 41/44 | 0.372 | 0.067 | 0.000076 | yes | yes | yes | yes | no | yes | 3 | fHbp+NHBA+PorA | 1b |
| 392 | 2011 | 10541 | 35 | 0.028 | 0.076 | 0.000016 | no | yes | no | yes | no | no | 1 | fHbp | - |
| 400 | 2011 | 10493 | 32 | 0.696 | 0 | 0 | no | yes | yes | no | no | no | 1 | NHBA | 3 |
| 401 | 2011 | 32 | 32 | 0.702 | 0.002 | 0 | no | yes | yes | no | no | no | 1 | NHBA | 3 |
| 404 | 2011 | 213 | 213 | 0.062 | 0.341 | 0 | no | yes | no | yes | no | no | 1 | fHbp | 7 |
| 405 | 2011 | 60 | 60 | 0.164 | 0.012 | 0 | no | no | no | no | no | no | 0 | no antigen | 7 |
| 406 | 2011 | 10864 | 269 | 0.289 | 0.013 | 0 | no | no | no | no | no | no | 0 | no antigen | - |
| 410 | 2010 | 146 | 41/44 | 0.195 | 0 | 0 | no | no | no | no | no | no | 0 | no antigen | 1a |
| 411 | 2010 | 33 | 32 | 0.715 | 1.082 | 0 | no | yes | yes | yes | no | no | 2 | NHBA+fHbp | 4 |
| 415 | 2010 | 269 | 269 | 0.43 | 0.013 | 0 | no | yes | yes | no | no | no | 1 | NHBA | 5 |
| 416 | 2010 | 154 | 41/44 | 1.11 | 0.03 | 0 | yes | yes | yes | yes | no | yes | 3 | fHbp+NHBA+PorA | 1b |
| 417 | 2010 | 32 | 32 | 0 | 0 | 0 | no | no | no | no | no | no | 0 | no antigen | 3 |
| 418 | 2010 | 146 | 41/44 | 0.267 | 0 | 0 | no | no | no | no | no | no | 0 | no antigen | 1a |
| 420 | 2009 | 10511 | 41/44 | 0.195 | 0 | 0 | no | no | no | no | no | no | 0 | no antigen | 1a |
| 421 | 2009 | 1163 | 269 | 0.267 | 0 | 0 | no | no | no | no | no | no | 0 | no antigen | - |
| 422 | 2009 | 146 | 41/44 | 0.123 | 0 | 0 | no | no | no | no | no | no | 0 | no antigen | 1a |
| 433 | 2009 | 318 | 41/44 | 1.243 | 0.014 | 0 | no | yes | yes | no | no | no | 1 | NHBA | 2 |
| 435 | 2009 | 32 | 32 | 0.437 | 0 | 0 | no | yes | yes | no | no | no | 1 | NHBA | 3 |
| 436 | 2009 | 575 | 213 | 0.119 | 0.012 | 0 | no | no | no | no | no | no | 0 | no antigen | 7 |
| 437 | 2009 | 3072 | 35 | 0.262 | 0.002 | 0 | no | no | no | no | no | no | 0 | no antigen | - |
| 438 | 2008 | 32 | 32 | 0.147 | 0 | 0 | no | no | no | no | no | no | 0 | no antigen | 4 |
| 439 | 2008 | 213 | 213 | 0.016 | 0 | 0 | no | no | no | no | no | no | 0 | no antigen | 7 |
| 440 | 2008 | 146 | 41/44 | 0.275 | 0.00022 | 0 | no | no | no | no | no | no | 0 | no antigen | 1a |
| 441 | 2008 | 1214 | 269 | 0.334 | 0.008 | 0 | no | yes | yes | no | no | no | 1 | NHBA | 5 |
| 442 | 2008 | 6058 | 41/44 | 0.803 | 0 | 0 | no | yes | yes | no | no | no | 1 | NHBA | - |
| 443 | 2008 | 213 | 213 | 0.014 | 0 | 0 | no | no | no | no | no | no | 0 | no antigen | 7 |
| 444 | 2008 | 318 | 41/44 | 0.302 | 0.068 | 0 | yes | yes | yes | yes | no | yes | 3 | fHbp+NHBA+PorA | 1b |
| 445 | 2008 | 10509 | 41/44 | 0.07 | 0.064 | 0 | no | yes | no | yes | no | no | 1 | fHbp | 1a |
| 446 | 2008 | 146 | 41/44 | 0.124 | 0.00041 | 0 | no | no | no | no | no | no | 0 | no antigen | 1a |
| 447 | 2008 | 13 | 269 | 0.079 | 0.00076 | 0 | no | no | no | no | no | no | 0 | no antigen | - |
| 448 | 2008 | 2166 | 269 | 0.43 | 0.017 | 0 | no | yes | yes | no | no | no | 1 | NHBA | 5 |
| 449 | 2007 | 318 | 41/44 | 0.568 | 0.031 | 0 | no | yes | yes | yes | no | no | 2 | NHBA+fHbp | 2 |
| 450 | 2007 | 318 | 41/44 | 0.264 | 0.018 | 0 | no | no | no | no | no | no | 0 | no antigen | 2 |
| 451 | 2007 | 136 | 41/44 | 0.212 | 0 | 0 | no | no | no | no | no | no | 0 | no antigen | - |
| 452 | 2008 | 461 | 461 | 0.595 | 0 | 0.000057 | no | yes | yes | no | no | no | 1 | NHBA | 6 |
| 453 | 2008 | 10511 | 41/44 | 0.154 | 0 | 0.0001 | no | no | no | no | no | no | 0 | no antigen | 1a |
| 454 | 2008 | 33 | 32 | 0.075 | 1 | 0.005 | yes | yes | no | yes | no | yes | 2 | fHbp+PorA | 3 |
| 455 | 2008 | 162 | 162 | 0.099 | 0.99 | 0.000068 | no | yes | no | yes | no | no | 1 | fHbp | - |
| 458 | 2007 | 146 | 41/44 | 0.16 | 0.0004 | 0.000096 | no | no | no | no | no | no | 0 | no antigen | 1a |
| 459 | 2007 | 10572 | 32 | 0.199 | 0.17 | 0 | no | yes | no | yes | no | no | 1 | fHbp | 4 |

** Isolates were estimated to be covered using MATS if the RP values were higher than 0.021, 0.294 and 0.009 for fHbp, NHBA and NadA, respectively. All isolates possessing P1.4 subfamily PorA were predicted to be covered.
